# Supplementary figures and images for: Settlement, environment, and climate change in SW Anatolia: Dynamics of regional variation and the end of Antiquity
Source: PLoS One. 2022 Jun 27;17(6):e0270295. doi: 10.1371/journal.pone.0270295 (PMC9236232; doi:10.1371/journal.pone.0270295)

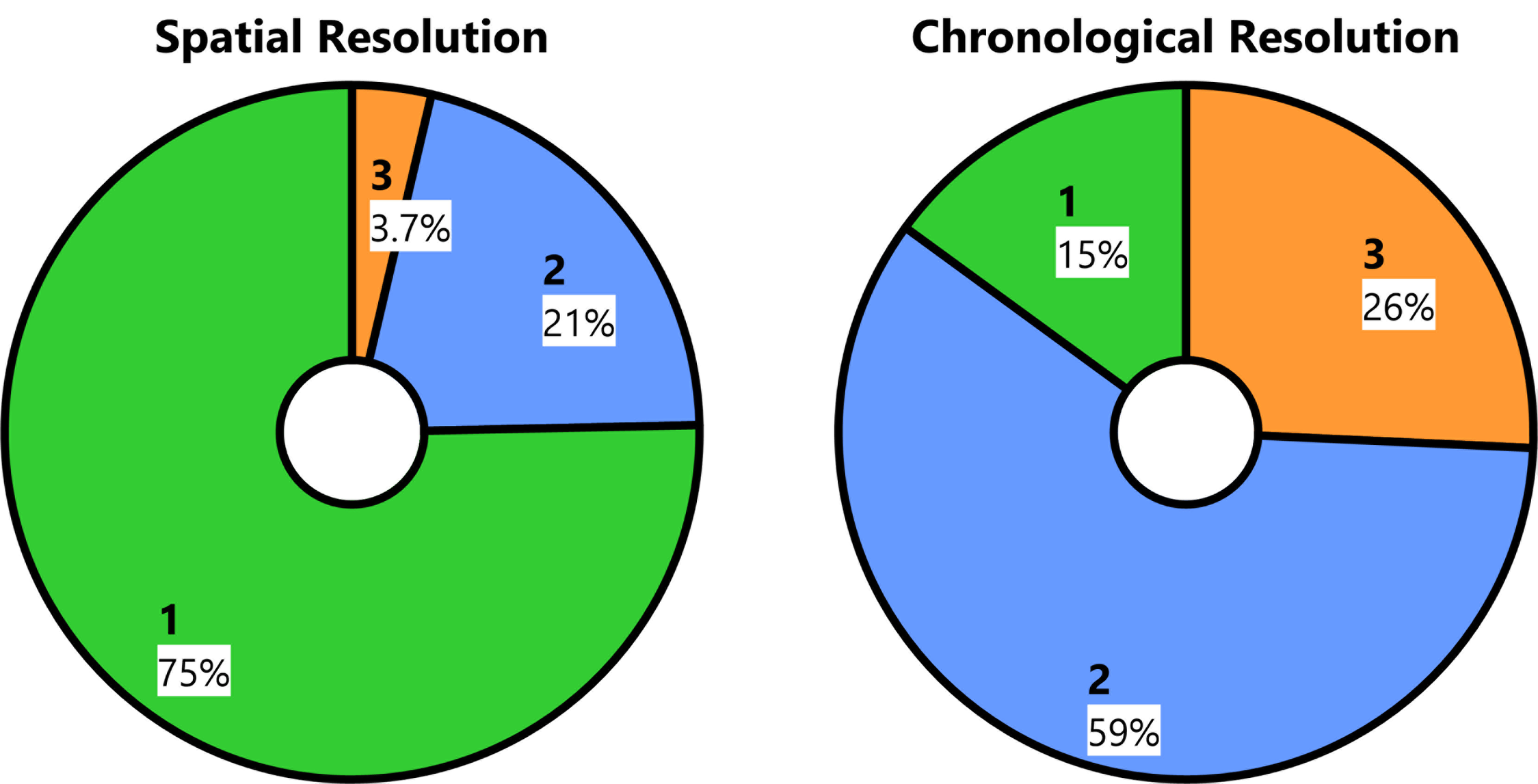

Supplement: S1 Fig — (TIF) [file pone.0270295.s001.tif]

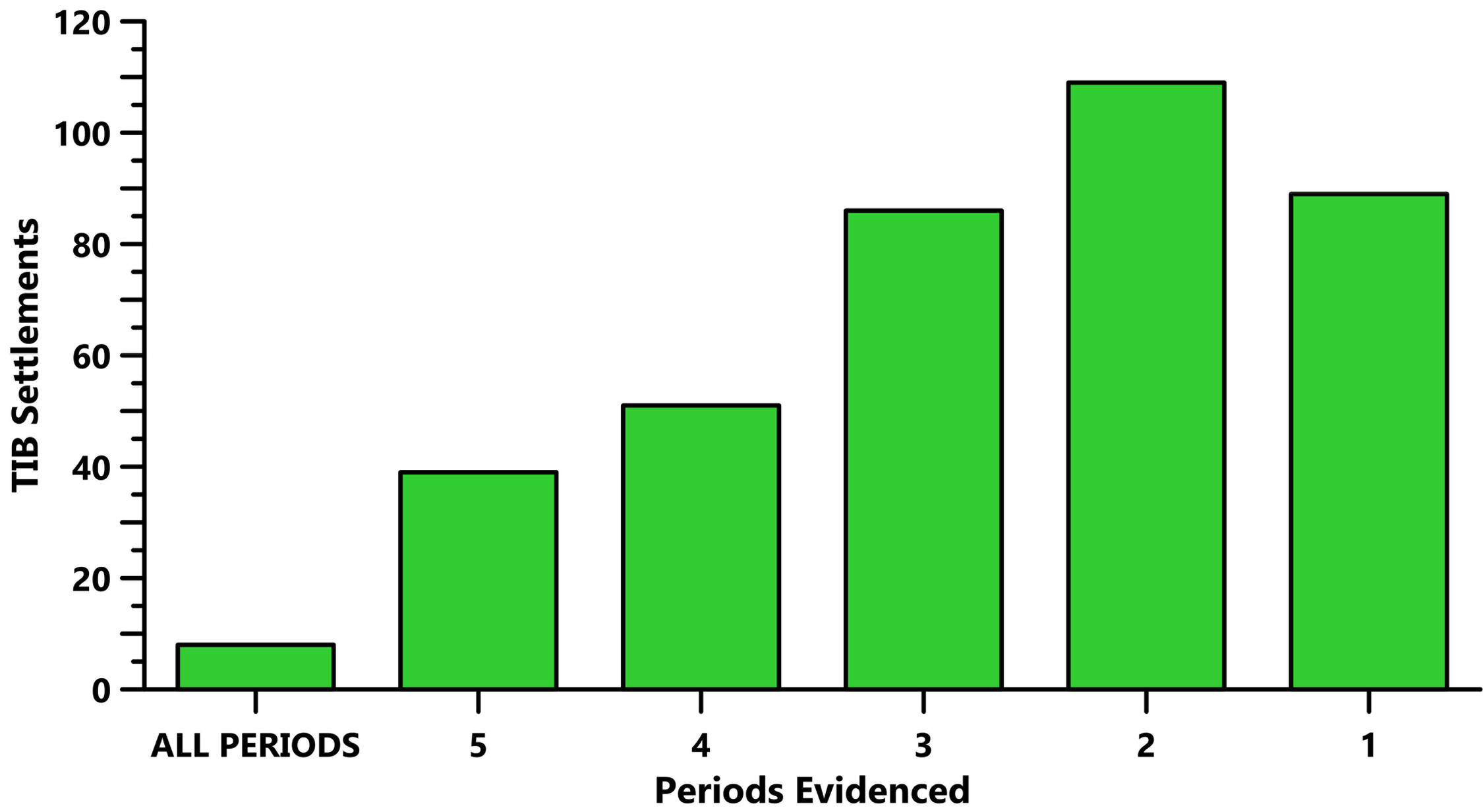

Supplement: S2 Fig — (TIF) [file pone.0270295.s002.tif]

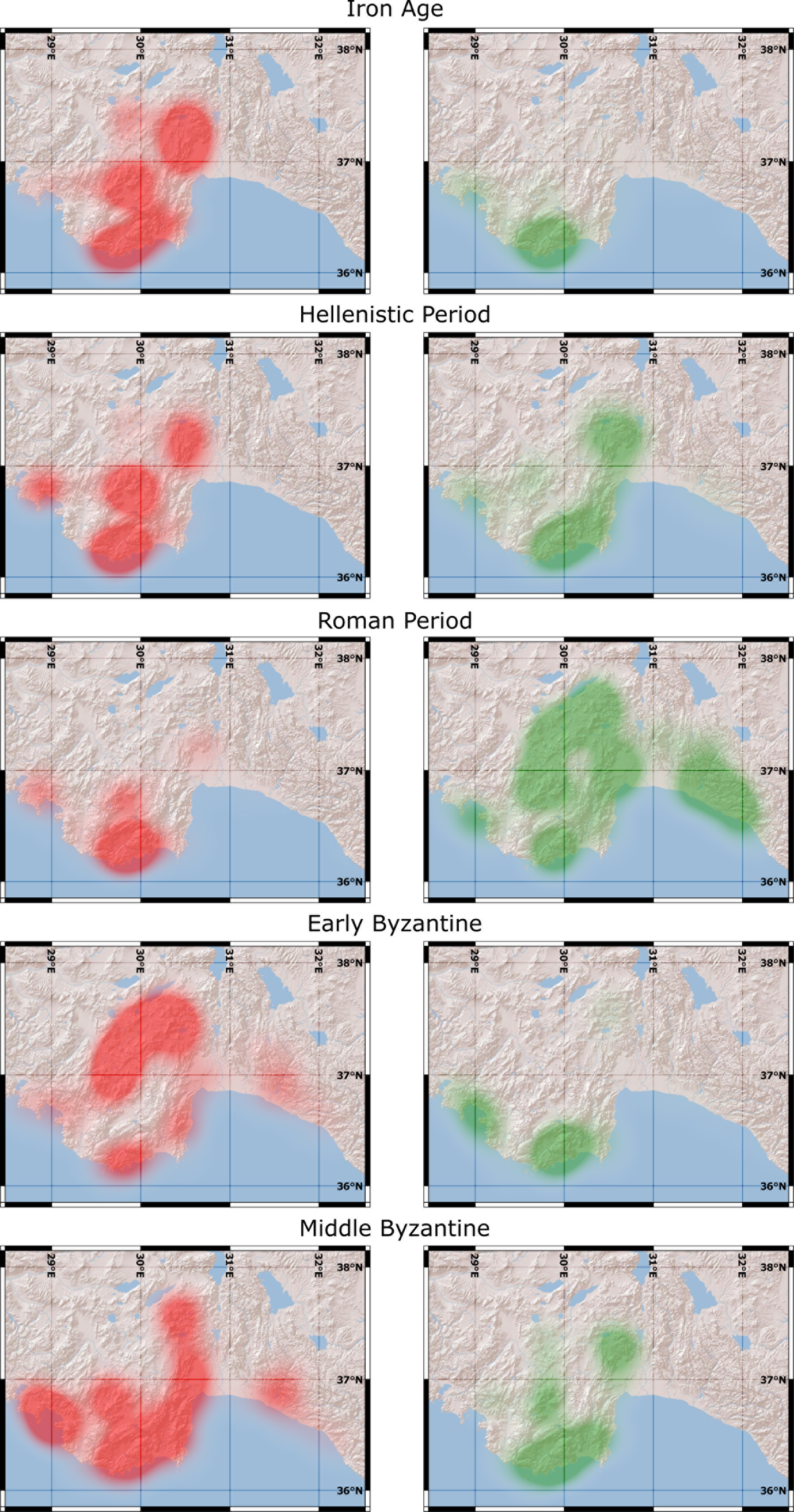

Supplement: S3 Fig — (TIF) [file pone.0270295.s003.tif]

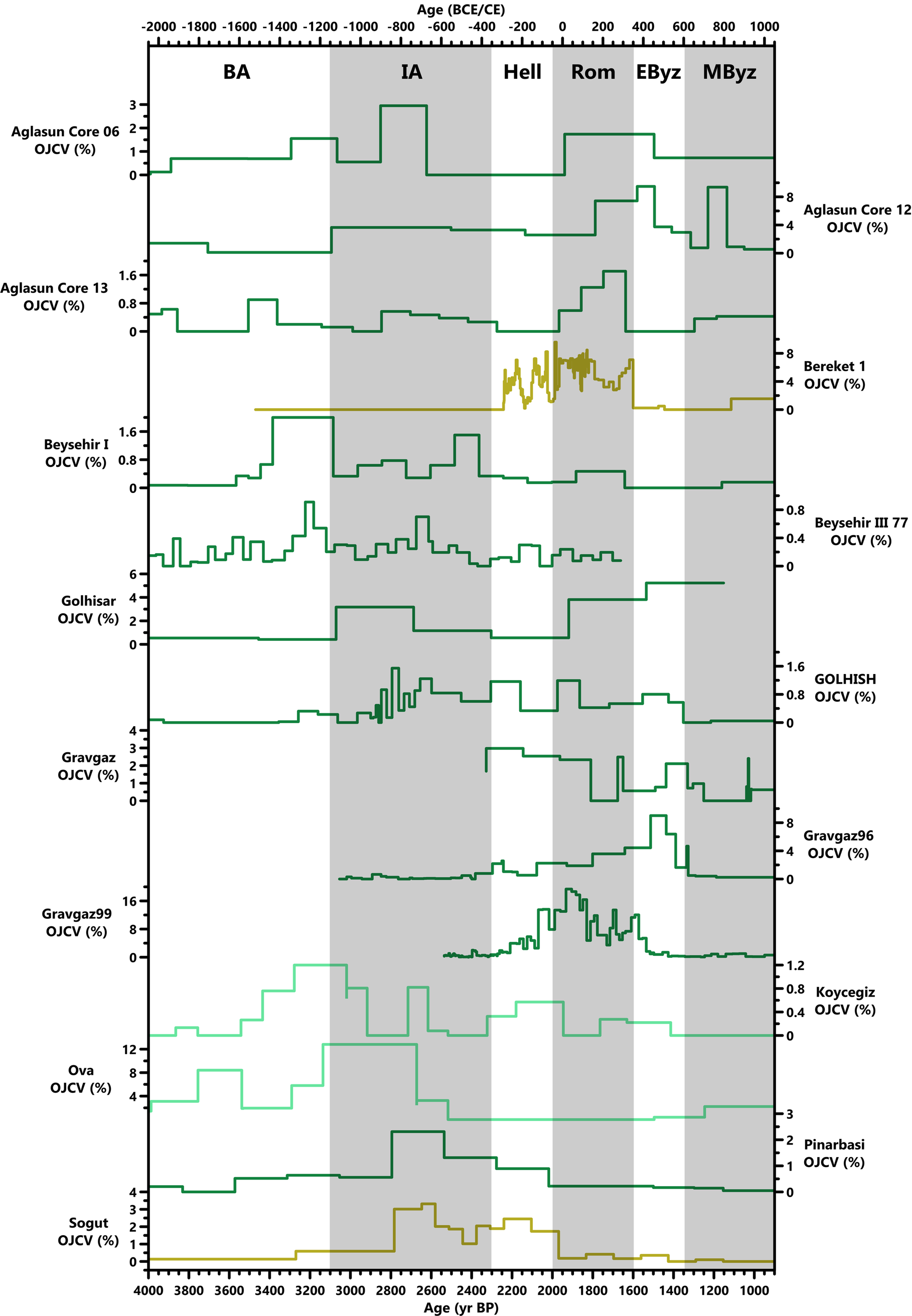

Supplement: S4 Fig — (TIF) [file pone.0270295.s004.tif]
