# Supplementary material for: Settlement, environment, and climate change in SW Anatolia: Dynamics of regional variation and the end of Antiquity
Source: PLoS One. 2022 Jun 27;17(6):e0270295. doi: 10.1371/journal.pone.0270295 (PMC9236232; doi:10.1371/journal.pone.0270295)
Supplement: Attachment — Submitted filename: PONE-D-22-00644_reviewer (1).pdf [file pone.0270295.s009.pdf]

PLOS ONE

Settlement, environment, and climate change in SW Anatolia: dynamics of regional variation and the end of Antiquity

--Manuscript Draft--

**Manuscript Number:**	PONE-D-22-00644
**Article Type:**	Research Article
**Full Title:**	Settlement, environment, and climate change in SW Anatolia: dynamics of regional variation and the end of Antiquity
**Short Title:**	Settlement and climate change in SW Anatolia
**Corresponding Author:**	Matthew J. Jacobson, BSc, MSc<br>University of Reading<br>Reading, Berkshire UNITED KINGDOM
**Keywords:**	archaeology; late antiquity; speleothems; climate change; history of climate and society; Anatolia; settlements; roman climatic optimum, late antique little ice age
**Abstract:**	In this paper we develop a regional dataset of change at 381 settlements for Lycia-Pamphylia in southwest Anatolia (Turkey) from volume 8 of the Tabula Imperii Byzantini – a compilation of historical toponyms and archaeological evidence. This region is rich in archaeological remains and high-quality palaeo-climatic and -environmental archives. Our archaeological synthesis enables direct comparison of these datasets to discuss current hypotheses of climate impacts on historical societies. A Roman Climatic Optimum, characterised by warmer and wetter conditions, facilitating Roman expansion in the 1 st -2 nd centuries CE cannot be supported here, as Early Byzantine settlement did not benefit from enhanced precipitation in the 4 th -6 th centuries CE as often supposed. However, widespread settlement decline in a period with challenging archaeological chronologies (c. 550-650 CE) is likely caused by a "perfect storm" of environmental, climatic, seismic, pathogenic and socio-economic factors, though a shift to drier conditions from c. 460 CE appears to have preceded other factors by at least a century.
**Order of Authors:**	Matthew J. Jacobson, BSc, MSc
	Jordan Pickett
	Alison L. Gascoigne
	Dominik Fleitmann
	Hugh Elton
**Additional Information:**	
**Question**	**Response**
**Financial Disclosure**<br><br>Enter a financial disclosure statement that describes the sources of funding for the work included in this submission. Review the submission guidelines for detailed requirements. View published research articles from *PLOS ONE* for specific examples.<br><br>This statement is required for submission and **will appear in the published article** if the submission is accepted. Please make sure it is accurate.	This work was supported by the AHRC South, West and Wales Doctoral Training Partnership (Grant AH/L503939/1 to M. J. Jacobson). The funders had no role in study design, data collection and analysis, decision to publish, or preparation of the manuscript.

Powered by Editorial Manager® and ProduXion Manager® from Aries Systems Corporation

**Unfunded studies**<br>Enter: *The author(s) received no specific funding for this work.*<br><br>**Funded studies**<br>Enter a statement with the following details:<br>• Initials of the authors who received each award<br>• Grant numbers awarded to each author<br>• The full name of each funder<br>• URL of each funder website<br>• Did the sponsors or funders play any role in the study design, data collection and analysis, decision to publish, or preparation of the manuscript?<br>• **NO** - Include this sentence at the end of your statement: *The funders had no role in study design, data collection and analysis, decision to publish, or preparation of the manuscript.*<br>• **YES** - Specify the role(s) played.<br><br>* typeset	
**Competing Interests**<br><br>Use the instructions below to enter a competing interest statement for this submission. On behalf of all authors, disclose any competing interests that could be perceived to bias this work—acknowledging all financial support and any other relevant financial or non-financial competing interests.<br><br>This statement is required for submission and **will appear in the published article** if the submission is accepted. Please make sure it is accurate and that any funding sources listed in your Funding Information later in the submission form are also declared in your Financial Disclosure statement.<br><br>View published research articles from *PLOS ONE* for specific examples.	The authors have declared that no competing interests exist.

**NO authors have competing interests**<br><br>Enter: *The authors have declared that no competing interests exist.*<br><br>**Authors with competing interests**<br><br>Enter competing interest details beginning with this statement:<br><br>*I have read the journal's policy and the authors of this manuscript have the following competing interests: [insert competing interests here]*<br><br>* typeset	
**Ethics Statement**<br><br>Enter an ethics statement for this submission. This statement is required if the study involved:<br><br>• Human participants<br>• Human specimens or tissue<br>• Vertebrate animals or cephalopods<br>• Vertebrate embryos or tissues<br>• Field research<br><br>Write "N/A" if the submission does not require an ethics statement.<br><br>General guidance is provided below. Consult the submission guidelines for detailed instructions. **Make sure that all information entered here is included in the Methods section of the manuscript.**	N/a

**Format for specific study types**<br><br>**Human Subject Research (involving human participants and/or tissue)**<br>• Give the name of the institutional review board or ethics committee that approved the study<br>• Include the approval number and/or a statement indicating approval of this research<br>• Indicate the form of consent obtained (written/oral) or the reason that consent was not obtained (e.g. the data were analyzed anonymously)<br><br>**Animal Research (involving vertebrate animals, embryos or tissues)**<br>• Provide the name of the Institutional Animal Care and Use Committee (IACUC) or other relevant ethics board that reviewed the study protocol, and indicate whether they approved this research or granted a formal waiver of ethical approval<br>• Include an approval number if one was obtained<br>• If the study involved *non-human primates*, add *additional details* about animal welfare and steps taken to ameliorate suffering<br>• If anesthesia, euthanasia, or any kind of animal sacrifice is part of the study, include briefly which substances and/or methods were applied<br><br>**Field Research**<br><br>Include the following details if this study involves the collection of plant, animal, or other materials from a natural setting:<br>• Field permit number<br>• Name of the institution or relevant body that granted permission	
**Data Availability**<br><br>Authors are required to make all data underlying the findings described fully available, without restriction, and from the time of publication. PLOS allows rare exceptions to address legal and ethical concerns. See the PLOS Data Policy and FAQ for detailed information.	Yes - all data are fully available without restriction

A Data Availability Statement describing where the data can be found is required at submission. Your answers to this question constitute the Data Availability Statement and **will be published in the article**, if accepted.<br><br>**Important:** Stating 'data available on request from the author' is not sufficient. If your data are only available upon request, select 'No' for the first question and explain your exceptional situation in the text box.<br><br>Do the authors confirm that all data underlying the findings described in their manuscript are fully available without restriction?	
**Describe where the data may be found in full sentences. If you are copying our sample text, replace any instances of XXX with the appropriate details.**<br><br>• If the data are **held or will be held in a public repository**, include URLs, accession numbers or DOIs. If this information will only be available after acceptance, indicate this by ticking the box below. For example: *All XXX files are available from the XXX database (accession number(s) XXX, XXX.)*.<br>• If the data are all contained **within the manuscript and/or Supporting Information files**, enter the following: *All relevant data are within the manuscript and its Supporting Information files.*<br>• If neither of these applies but you are able to provide **details of access elsewhere**, with or without limitations, please do so. For example:<br><br>*Data cannot be shared publicly because of [XXX]. Data are available from the XXX Institutional Data Access / Ethics Committee (contact via XXX) for researchers who meet the criteria for access to confidential data.*<br><br>*The data underlying the results presented in the study are available from (include the name of the third party*	All relevant data are within the manuscript and its Supporting Information files.

Powered by Editorial Manager® and ProduXion Manager® from Aries Systems Corporation

and contact information or URL).<br>• This text is appropriate if the data are owned by a third party and authors do not have permission to share the data.<br><br>* typeset	
Additional data availability information:	

Main MS File

Click here to access/download;Manuscript;Jacobson et al - Settlement and climate in SW Anatolia.docx

Settlement, environment, and climate change in SW Anatolia: dynamics of regional variation and the end of Antiquity

Matthew J. Jacobson[a, *], Jordan Pickett[b, *], Alison L. Gascoigne[c], Dominik Fleitmann[d], Hugh Elton[e]

[a] School of Archaeology, Geography and Environmental Science, University of Reading, Reading, RG6 6UR, UK

[b] Department of Classics, University of Georgia, 30602 Athens GA, USA

[c] Archaeology, University of Southampton, Southampton, SO17 1BJ, UK

[d] Department of Environmental Sciences, University of Basel, 4056 Basel, Switzerland

[e] Ancient Greek & Roman Studies, Trent University, Peterborough, ON, K9L 0G2, Canada

[*] Corresponding authors. E-mail address: m.j.jacobson2@pgr.reading.ac.uk (Matthew J. Jacobson) and jordan.pickett@uga.edu (Jordan Pickett)

Abstract: In this paper we develop a regional dataset of change at 381 settlements for Lycia-Pamphylia in southwest Anatolia (Turkey) from volume 8 of the *Tabula Imperii Byzantini* – a compilation of historical toponyms and archaeological evidence. This region is rich in archaeological remains and high-quality palaeo-climatic and -environmental archives. Our archaeological synthesis enables direct comparison of these datasets to discuss current hypotheses of climate impacts on historical societies. A Roman Climatic Optimum, characterised by warmer and wetter conditions, facilitating Roman expansion in the 1st-2nd centuries CE cannot be supported here, as Early Byzantine settlement did not benefit from enhanced precipitation in the 4th-6th centuries CE as often supposed. However, widespread settlement decline in a period with challenging archaeological chronologies (c. 550-650 CE) is likely caused by a "perfect storm" of environmental, climatic, seismic, pathogenic and

socio-economic factors, though a shift to drier conditions from c. 460 CE appears to have preceded other factors by at least a century.

Introduction

Past climatic and environmental conditions are often considered influential for socio-cultural change in Anatolia and the broad Eastern Mediterranean-Middle East region (e.g., [1–3]. Large-scale analyses are often necessitated, but also hindered, by low availability of high-quality comparable interdisciplinary datasets proximate to one-another (e.g., the Negev desert [4,5[illegible]his presents difficulties in analysis as both climatic and socio-economic conditions display high spatial and temporal variability [6–8]. Here, we develop a regional settlement change dataset for Lycia and Pamphylia in SW Turkey (Figure 1), adapted from volume 8 of the *Tabula Imperii Byzantini* (TIB 8: [9]) — a compilation of historical toponyms and associated archaeological research [10]. The region is rich in the archaeological remains of cities, harbours, and rural settlements, in addition to high-quality palaeo-climatic and -environmental datasets. Previously, the archaeological evidence was disjointed due to a lack of data-synthesis [11].

Figure 1: Maps of the study region: (**a**) All TIB 8 settlements compared to elevation. Important locations are named, ancient (modern) names: Xanthos (Letoon), Balboura (Çölkayiği), Myra (Demre), Attaleia (Antalya), Side (Manavgat). (**b**) Location of study region

within the Eastern Mediterranean. (**c**) Lycia-Pamphylia with palaeoclimate records (triangles, named) and pollen records (squares). Pollen records are: Köyceğiz (a), Ova (b), Sögüt (c), Pinarbaşı (d), Bereket Basin (e), Gravgaz Marsh (f), Ağlasun (g) and Beyşehir (h).

Following the production of our settlement dataset, available evidence in Lycia-Pamphylia meets a sufficient standard to test hypotheses linking climatic and socio-cultural change. Abundant palaeo-environmental datasets (15 pollen records, from 9 sites) located at varied altitudes (Figure 1c), detail past ecological conditions and the intensity of human agricultural activities [12–14]. Pollen in these records detail a period of human-induced land-cover change known as the Beyşehir Occupation Phase (BOP), characterised by reduced presence of forest taxa and increased presence of cultivated trees [15]. For analysis in this paper, we utilise the presence of cultivated trees, represented by OJCV (*Olea* (Olive), *Juglans* (Walnut), *Castanea* (Chestnut) and *Vitis* (Grapevine)) pollen, calculated as an average of standardised percentages for archaeological periods and overlapping 200-year time windows, as a proxy for anthropogenic influence/ intensity of agricultural activities [14,16]. The potential agricultural productivity of land is heavily determined by location (including elevation) and climatic factors. In SW Turkey, effective moisture (soil moisture available to plants) is the primary climatic constraint to plant growth and, therefore, agricultural productivity [17,18]. The recent publication of a highly-resolved speleothem (stalagmite) proxy record from Kocain Cave, ~38km north of Antalya enables detailed reconstruction of regional effective moisture and precipitation amount for >3,000 years [19]. Measured ratios of magnesium with calcium (Mg/Ca) record changes in effective moisture at a very high resolution (>1 sample per year). Variations in oxygen isotope ratios ($\delta^{18}O$) provide

information about past precipitation at decadal timescales (average of 9 years between samples). Two other palaeoclimate records are in our study region and cover the period under discussion. The lower resolution Lake Gölhisar $\delta^{18}O$ record [20], located within the Lycian Taurus mountains at 930m asl, reflects lake-water balance, a variable akin to effective moisture, on a sub-centennial timescale (average of 71 years between samples for 1,000 BCE – 1,050 CE). The multi-proxy Lake Salda record [21], located in Burdur Province at 1180m asl, extends back to ~560 CE and reflects decadal-scale changes (average of 12 years between samples for 560-1050 CE) in lake-water balance. These records detail high climatic variability during the Late Holocene, with numerous dry and wet phases identified.

Our record of settlement change is compared diachronically to these high-quality palaeo-climatic and -environmental datasets to examine the influence of climate change on settlement patterns. Based on this comparison, we argue that in our study region climate change does impact agricultural productivity, which can lead to changes in settlement density and locations. However, the relative impact is highly variable, and socio-political factors are often more important.

Study area geography

Our study region, covered by the TIB 8, pertains to the ancient regions of Lycia and Pamphylia, as well as parts of southern Pisidia, which are contained within 28°30′-32°30′E and 36°00′-38°00′N (Figure 1b). The Western Taurus mountains form an orographic barrier to southerly and south-westerly airflows, separating the coastal and interior plateau regions into zones with distinct microclimates, ecological conditions, and high numbers of endemic

species [22–24]. According to the Köppen-Geiger climate classification system, coastal regions are categorised as hot-summer Mediterranean (Csa) climates, with high levels of precipitation that exhibits a clear winter peak; inland regions are characterised by cold semi-arid (Bsk) and Mediterranean-influenced hot/warm-summer humid continental (Dsa/b) climates [25–27]. Colder conditions, with reduced precipitation (~33% less) characterise the regions further inland and at higher elevations [28,29]. Fertile river valleys (e.g., the Dalaman and Köprüçay) that cut through the mountains are ideal for both rain-fed and irrigated agriculture. These run from the elevated Anatolian plateau, which is dotted with lacustrine basins and upland sheep and goat pastures called yaylas, down to indented coastlines that provide convenient natural harbours for maritime trade [30–33].

Methods

We tabulated entries in Lycia-Pamphylia from the TIB 8 to create a record of settlement change between the Bronze Age and Middle Byzantine period. The TIB is a long-term project organised by the Austrian Academy of Sciences since 1966, which has coordinated historical and modern toponyms for provinces of the Roman-Byzantine Empire with an extensive compilation of primary historical sources for all periods and relevant languages, ancient inscriptions, traveller accounts from the 19th and 20th centuries, and published data from extensive archaeological surveys and excavations [10]. All toponyms in the TIB 8 that could be accurately located, with archaeological or primary source evidence that could be attributed to at least one of six periods (Table 1) were compiled and their locations and elevations were identified using satellite imagery and GIS. The history and

bibliography of each toponym was reviewed, including new publications since the TIB 8 was published in 2004, to attribute presence or absence of evidence to each period throughout c. 3000 BCE – 1050 CE. The periodisation of evidence is based on established historical periods. The type and nature of evidence from each site was also noted according to 5 categories, in order of spatial and chronological certainty (Tables 1 and 2):

Table 1: Measures of resolution (visually presented in Figure S1)

Measure of Resolution	Explanation	Number of Settlements
Spatial		
1	Site location <1km	291
2	Site location 1-3km	76
3	Site location >3km	14
Chronological		
1	Site has been excavated or systematically surveyed and published	56
2	Site has been extensively surveyed by scholars; often reliant on architectural description and broad period indicators	226

3	Site has been summarily visited by scholars; no formal excavation or survey; or, reused architectural elements of a given period noted in medieval-modern settlement	99

Table 2: Settlement evidence by period.

Period	Architectural	Material Culture	Negative Architectural	Literary Reference	Spolia
Bronze Age	3	20		2	0
Iron Age	63	30		19	2
Hellenistic	136	42		24	3
Roman	147	101		11	17
Early Byzantine	213	23		7	12
Middle Byzantine	45	2	72	10	6

1. New or restored architecture – including monumental and public or domestic structures, and fortifications.

2. Architectural contraction – pertaining to church contractions in the Middle Byzantine period only, where communities constructed a smaller chapel within the confines of an older church complex [34].

3. Material culture – mainly ceramics, also tombs and graves, coins and inscriptions located within settlements.

4. Textual reference – settlements referenced by ancient primary sources, whether in literature, or by inscriptions and mint marks of coins found outside the named settlement (e.g., the tribute lists of the Delian League).

5. Spolia – older construction materials that have been reused in ancient, medieval, or modern settlements. In large quantities from ancient and medieval contexts these portend earlier settlement at a site; settlements with limited quantities of spolia in early modern contexts that may have been transported for some distance have not been included.

Our study ends at the beginning of the Turkish conquests in the 11th century CE, when the region became politically fragmented and the nature of both archaeological and textual evidence changed radically [9,35]. Measures of spatial and chronological resolution were assigned to each settlement, based on confidence in its location and archaeological history, for the best represented period (Table 1).

Data Critique

Significant interpretative challenges are presented when producing a settlement dataset from historical toponyms. These are analogous to those well-understood for archaeological survey data ([36–39]; references below). Our dataset will reflect part of the original distribution of settlements, but understanding biases produced by chronological

challenges and varied preservation is important to determine the accuracy of the pattern. There are four main issues present:

Firstly, the six periods utilised in this study vary in length from 250 to 1850 years (Table 1) which will lead to overemphasis of settlement numbers during longer periods. Time-adjusted settlement numbers can also help to compensate for this bias, these are calculated (following the methodology of [37,40]) as follows:

$$Time\ adjusted\ settlement\ number = \frac{Total\ settlement\ number}{(Period\ length/Shortest\ period\ length)}$$

The shortest period length in our study is the Early Byzantine Period (250 years). In our discussion, we focus on periods with relatively short and similar durations.

Secondly, categorising settlements into periods by relative dating (periodisation/ "time-averaging") brings inherent bias. This makes settlements appear to exist for the entire period (the "synchronistic paradigm"; [41]). Thus, they are only given a *terminus ante* and *post quem* at the start and end of the archaeological period. For many settlements, particularly rural, it is impossible to tell whether they were occupied throughout a particular period, for a short duration, or abandoned and reoccupied [42]. Settlements can therefore appear contemporaneous though they were not (the "contemporaneity problem"; [43,44]). The precise definition of chronological boundaries also obscures the uncertainty associated with archaeological chronologies, which rarely correspond perfectly to one-another. For instance, the Lycian-type tombs and sarcophagi that dot the landscape remain little changed between the Late Iron Age into the Early Roman period. Different strategies and resolutions

for periodisation are utilised in different academic disciplines, such as by ceramicists when compared to historians.

Thirdly, destruction of evidence in later periods and time-dependent degradation will lead to an overemphasis of settlement numbers in more recent periods and alter the settlement pattern via a "preservation bias" [45]. Evidence in a particular period determines its archaeological visibility. Standing architectural remains, which constitute the bulk of archaeological evidence in our study region since the Iron Age, have an exceptional degree of preservation in Lycia-Pamphylia due to the region's relative inaccessibility and low population density. Preservation has contributed to a long history of archaeological exploration (e.g., [46]) via extensive survey and architectural description, at the expense of excavated ceramics whose publication is more limited. Ceramics are generally robust, but different raw materials and production processes determine the level of survival, and visibility, that can change the number of sherds surviving from each period: red-slip wares from the Late Hellenistic to Late Roman periods are better represented in publication, for instance [47]. In addition to the archaeological taphonomy of settlements, biases impacting the survival of ancient site toponyms may influence our record. Whilst we include some settlements with archaeological remains that can only be identified by a modern Turkish toponym, ancient toponym survival is comparatively strong in our study region, caused by the continued presence of Greek communities until population exchanges after 1923 [48]. However, the literary and historical record does yield additional ancient toponyms that cannot be securely located on the landscape, and there are likely many smaller settlements whose toponyms did not survive, left no evidence, or were subsequently destroyed.

Finally, indications of settlement or population size, whose estimates are typically dependent on measurable area within city walls or counts of housing units [49], are beyond the scope of this paper. A related problem is the nature of the historical *polis*, commonly translated as "city". The polis was a rank of settlement since ~600 BCE that signalled the presence of municipal government and elite investment in monumental architecture [50]. However, such settlements did not necessarily have high-density populations and most Lycian-Pamphylian cities lack substantial domestic architecture and never reached the size, density, or population, of Ephesos, Antioch, or even Tarsus [51,52]. In the Early Byzantine period the rank of polis was extended to more settlements, even as agrarian and artisanal-industrial activities were introduced within city walls [53](see Discussion). In the Middle Byzantine period, many *poleis* retained their status even in cases where the archaeological evidence suggests they were small villages (e.g., Pinara or Pednelissos; see Discussion). The extent to which Lycian-Pamphylian *poleis* or "cities" may be classified as low- or high-density urban environments is therefore irresolvable in many cases [54]. In our metadata, increasing settlement density across the study region could therefore represent expansion or fragmentation, with associated population increases or decreases. Settlement types, so far as they can be reconstructed, are included in our full data (Table S1).

Results

Within our study area, 381 out of the total 1038 toponym entries in TIB 8 could be located and identified as a settlement, with sufficient survival or publication to enable identification of historical presence between the Bronze Age and the Middle Byzantine period (Table 2). The remaining 658 entries were either duplicates, dated to later periods

only, infrastructure not associated with settlement (e.g., bridges), natural phenomena (e.g., rivers, mountains), regional toponyms, or their location was unknown. Toponyms associated with settlements are varied, including cities, farmsteads, peri-urban harbours and temples, hilltop fortifications, island villages and caves. Settlement function, size, and status changed over time. Of 381 settlements, only 8 were occupied in all six periods and 283 or ¾ of the total contained evidence for 1-3 periods (Figure S2). Elevation of settlements varied overall, ranging from 0 to 1835m asl, and by period (Figures 2 and 5). However, lower elevation settlements dominate, with 29.4% at elevations <200m asl and 70% at elevations <1,000m asl. Most settlements (291) could be located to <1km and only a very small portion (14) could not be located to <3km (Table 2). Chronological resolution of settlements was weaker, with only 56 excavated or systematically surveyed and published. A total of 226 settlements were extensively surveyed but with reliance on rather broad and primarily architectural indications for chronology. The remaining 99 have weak chronological resolutions, being only summarily visited by travellers or scholars since 1800 CE, or dated by re-used building materials, easily recognisable within later buildings (e.g., Classical architectural sculpture or inscriptions recycled in Late Roman fortifications). The character of settlement evidence also varied by period (Figures 2c and 3).

Table 3: Settlement metadata by period. “New” settlements are those with evidence in the period, but no evidence in the preceding period; “Continued” settlements are those with evidence in the period and preceding period; “Abandoned” settlements are those with no evidence in the period, but evidence in the preceding period; time-adjusted settlement

numbers are calculated following the methodology of Contreras et al., (2018) and Barton et al., (1994).

Period	Length (years)	Total Settlements	New Settlements	Continued Settlements	Abandoned Settlements	Time-Adjusted	Avg. Elevation (m asl)
Bronze Age (3000 – 1150 BCE)	1850	25	25	N/a	N/a	3.38	759
Iron Age (1150 – 350 BCE)	800	114	100	14	11	35.63	554
Hellenistic (350 – 50 BCE)	300	205	104	101	13	170.83	615
Roman (50 BCE – 350 CE)	400	276	116	160	45	172.5	664
Early Byzantine (350 – 600 CE)	250	255	66	190	87	255	575
Middle Byzantine (600 – 1050 CE)	450	135	17	118	137	75	562

Figure 2: Presentation of the TIB data. (**a**) Total settlement data, (**b**) time-adjusted data (**c**) settlement numbers by evidence type.

Figure 3: Maps showing settlement evidence by period.

Figure 4: Photographs and plans from our study region by Jordan Pickett, excluding 6.d. Photographs and plans from study region by Jordan Pickett, excluding 6.d. (a) A Lycian alluvial landscape, looking west over the river Eşen Çayı / anc. Xanthos from 7th-9th century Byzantine fortifications on the Lycian Acropolis at city of Xanthos, with modern greenhouses below; (b) the Roman colonnaded street with cascading fountains fed by aqueduct at Perge; (c) Sixth century CE architectural sculpture from church at Asarcık; (d) Tlos, with Roman stadium in foreground, Iron Age-Hellenistic sarcophagi and tombs in mid-ground, and Early-Middle Byzantine fortifications built atop the Iron Age acropolis (photo under Creative Commons License from flickr user sarah_c_murray); (e) Annotated satellite image of Xanthos, with Lycian Acropolis (since 8th BC), Iron Age fortifications (in orange), late Classical and Hellenistic city grid with agorai at east, Roman agora and theater at west, Early Byzantine basilicas, and Middle Byzantine refortification of the Iron Age Lycian Acropolis (in red); (f) plan of Middle Byzantine contraction of older church basilica on Xanthos Acropolis (by Jordan Pickett, after Erdoğan and Ceylan 2019).

Interpreting changes in the number and locations of settlements for each period presents significant challenges, associated with chronology, interpretive uncertainty, and

preservation bias (see Data Critique). However, some patterns are still observable and broadly consistent with the regional history, archaeological evidence, and palaeoenvironmental data. The clearest of these trends is a steady increase and peak of settlement numbers in the Roman and Early Byzantine periods, followed by a significant reduction of Middle Byzantine evidence (Figures 2a and 3). These changes are consistent with data from across the Eastern Mediterranean [1,55,56] and are frequently hypothesised to result in part from changing climatic and environmental conditions. We discuss these hypotheses below, utilising a regional case-study approach that includes comparison of our dataset with pre-existing evidence.

Figure 5: Settlement elevation data by period. Box-plots with inter-quartile ranges and median values (top) and mean elevations (bottom).

Discussion

History of Climate and Society in the Eastern Mediterranean

Studying associations between climatic and socio-cultural change, now termed the 'history of climate and society' (HCS; [57]) in the Eastern Mediterranean has been extensive, due to the region's historical significance, and a focus of many popular-science books [58–60]. Generally, such studies produce climate reconstructions which can be, linked to large-scale climatic periods or events (such as those displayed in Figure 6c), from a combination of palaeoclimate proxy records, model simulations, and historical records. Assembled climate evidence is then compared to historical and archaeological evidence for social, economic,

and cultural change, to assess human-climate-environment relationships. These studies intend to provide insight into past societal resilience and fragility, to prepare for potential impacts of future climate change [8,61]. However, reviews of HCS studies [57,62–65] have identified significant and recurring issues, namely (**1**) correlation-based conclusions that lack convincing causal explanations, (**2**) a bias towards periods of “crisis” which mischaracterises human-environment interactions, (**3**) a focus on large regions without high-quality comparative datasets, leading to calls for “micro-regional” case-studies (stressed originally in [6]), and (**4**) the interdisciplinary challenge of comparing archaeological, palaeo-environmental, and -climatic data of varied resolutions. These challenges are confronted in our presentation of regional data for climate and society in Lycia-Pamphylia.

Figure 6: Regional palaeoclimate data (Table S2) compared to (a) Mg/Ca averages from Ko-1 and (b) $\delta^{18}O$ from Gölhisar and Ko-1. (c) Full palaeoclimate data with warm/cold intervals: Crisis Years Cold Event (CYCE), Roman Climatic Optimum (RCO), Dark Ages Cold Period (DACP), Late Antique Little Ice Age (LALIA) and Medieval Climate Anomaly (MCA). Kocain Mg/Ca, Gölhisar $\delta^{18}O$, and Salda Ca/Fe, are largely indicative of effective moisture; Kocain $\delta^{18}O$ reflects precipitation, with secondary influences from seasonality and temperature [19–21].

HCS studies focused on the Eastern Mediterranean are numerous, with climate change frequently presented as an important factor driving prosperity and decline. In the sections below, we critique this over-simplified and generalised model, by examining three previous hypotheses. Firstly, the Roman Climatic Optimum (RCO; previously Roman Warm

Period) is a proposed period of warm and wet conditions controversially referenced as a driving force behind the large-scale success of the Roman Empire [60]. Advantageous climatic conditions are suggested to have contributed to enhanced agricultural productivity, with associated economic, demographic, and settlement growth, between 200 BCE and 150 CE [60]. The 1st and 2nd centuries CE have long been suggested as a period of unprecedented prosperity (the "Pax Romana") – a concept recently critiqued and discussed in [66,67]. Secondly, enhanced precipitation has been designated as an enabling factor for Central and Eastern Mediterranean prosperity in the 4th- early 6th centuries CE [1,68–70]. During this period, increased interconnectivity between regions, higher agricultural productivity, population, and settlement numbers, alongside the expansion of rural settlement onto previously unoccupied marginal land, are suggested [52]. Thirdly, a period of cooling and/or drying, beginning in the 6th century CE, has been suggested as an important contributing factor for a decline across the Eastern Mediterranean and surrounding regions in the 6th and 7th centuries CE [71–73]. Epidemics and famines are theorised to have been caused by climatic deterioration, leading to demographic contraction and settlement abandonment through deaths and migration.

There are several key criticisms of these types of hypotheses, linked to the above broader criticisms of HCS studies. Most apparent is the generalised and large-scale nature of such hypotheses. Thus, care must be taken when discussing generalised patterns for broad regions or periods, as both climatic and socio-economic conditions have the potential for high variability on both spatial and temporal scales, especially in the Eastern Mediterranean [6–8,11,19]. Regional analyses are therefore required where the main climatic constraints on agricultural productivity are identified. For most regions in the Eastern Mediterranean, the

basic assumption that higher temperatures and water availability will facilitate higher agricultural productivity is too simplistic [3,17]. A regional scale analysis of Lycia-Pamphylia is enabled by our new dataset of settlement character and location, and the existing high quality palaeo-environmental and -climatic records. Chronological and preservation biases in settlement datasets are limiting factors for this analysis (see Data Critique). However, the consistency of patterns across varied data-types, particularly for the Roman to Middle Byzantine periods, suggests they result from regional changes in settlement density and distribution. Comparing datasets of different temporal resolutions, such as highly-resolved palaeoclimate records and period-averaged settlement data, presents significant challenges. Averaging of high-resolution palaeoclimate data for archaeological periods or centuries is one methodology frequently utilised in the Eastern Mediterranean (e.g., [2,56,74]), here providing interesting results (Figures 6a and 6b). A visual relationship between $\delta^{18}O$ and settlement evidence is apparent (Figure 6b). However, century-long averages are misleading, and conceal the high variability of climatic conditions within each period (Figure 6c). Additionally, agricultural productivity is more reliant on effective moisture, which for the higher resolution Kocain Mg/Ca record [19] shows a weaker relationship (note the Roman Period in Figure 6a). In the sections below, we consider the full picture of climatic variability during the periods under discussion and assess the extent of their impact on regional settlement location and character, in addition to the contribution of other factors.

A Roman Climatic Optimum?

Roman settlements in our dataset were largely continuous with the preceding Hellenistic period. A total of 45 Hellenistic settlements were abandoned and the remaining

160 constitute 58% of the 276 Roman period settlements 50 BCE-350 CE. A shift in elevations is observed, with the highest mean elevation of all considered periods for new (736m asl) and total (664m asl) settlements (Figure 5). Abandoned settlements are more frequent at low elevations (37/45<1000m asl; mean=622m asl) in south central and western Lycia, especially in association with older Iron Age and Hellenistic Lycian dynastic centers. For example, within 20km of Myra and Finike (ancient Phoinix), at least 10 settlements contain Hellenistic fortified tower-farmsteads or "turmgehöfte" [75] that show no signs of Roman occupation, though many were reoccupied in the drier Early Byzantine period (e.g., Beymelek, Davazlar, Gelemen or Pharroa). Of all new Roman settlements, a significant portion are located at mid-high elevations (68/116>600m asl), with highland concentrations in Pamphylia north of Side and Alanya and in northern Lycia/south Pisidia (Figure S3). In the latter region, many rural Roman settlements are identified, primarily via inscriptions and ceramics, in surveys conducted by the Sagalassos Archaeological Research Project [76,77]. Rural settlements constitute a large proportion of new Roman settlements (63 villages and farmsteads). Industrial rural estates based on the Italian villa rustica model appear at 10 settlements (e.g., at Çaylakkale, Gedelma, Hamaxia). Conversely, most Roman period urban settlements (70/81) were continuations from the Hellenistic period.

Pollen data from around Sagalassos suggest that interior regions with high Roman period settlement density in our dataset (and in the Sagalassos Hinterland survey [78]; Figure 7) experienced expansion of upland agriculture and arboriculture in the 1st century CE. At the high-elevation Bereket Basin site, OJCV values peak with significant increases also occurring at Ağlasun, Gölhisar, and Gravgaz [79]; Figures 7 and S4). Despite proliferation of imperial period architecture and material culture, and expansion of agricultural activity that matches

with the RCO hypothesis, current evidence does not suggest that these were determined (or enabled) by climatic factors.

Figure 7: TIB settlement data and mean elevation by period compared to other regional surveys and OJCV value z-scores – per period and as 200-year averages. References found in the main text.

During the RCO, Mg/Ca ratios from Kocain Cave indicate that effective moisture was declining from ~100 BCE until ~30 BCE, then remained low until ~100 CE, with a further short-lived dry phase at ~220 CE also evidenced by a soot layer and pollen statistical analysis at Gravgaz Marsh [19,80,81]. Between these dry phases, Mg/Ca ratios were close to their mean values (0.88mmol/mol^{-1}) until ~120 CE and then lower (indicating slightly wetter conditions) until ~200 CE. $\delta^{18}O$ values from both Kocain Cave and Lake Gölhisar suggest slightly wetter conditions from 50/1 BCE; however, this may result from the lower-resolution of these archives which misses out shorter-term changes in climate. Slightly enhanced precipitation is suggested by $\delta^{18}O$ from Kocain Cave during 150-200 CE, matching the wetter conditions suggested by Mg/Ca. This likely reflects conditions wetter than the average for the Roman period, but not as wet as the start of the preceding Hellenistic Period or the distinct period of high effective moisture in the 4th and 5th centuries CE (~330-460 CE; [19]). In our study region, during the RCO, and Roman Period, there was a high level of variability, with little evidence to suggest an enduring warm and wet phase. In fact, a lack of evidence for consistently warmer and/or wetter conditions during the RCO is observed throughout the Eastern Mediterranean [74,82]. However, highly-resolved and well-dated palaeo-temperature records are more sparsely distributed in the Eastern Mediterranean, and rarely extend back

into the first millennium CE [8]. For example, the only palaeo-temperature record in Lycia-Pamphylia starts in 1125 CE [83]. Further work is needed to reveal whether temperature changes influenced the previously described settlement shifts.

At least in our study region, hydroclimatic conditions were not the main factor responsible for the expansion of agricultural activity and settlement during the Roman period. Instead, we argue that integration of the region into the Roman imperial system after ~50 BCE was more important. A formal treaty of Lycia with Caesar was signed in 46 BCE [84], under Augustus (27 BCE – 14 CE) the first colonies were established (e.g., at Balboura, Kremna, and Olbasa), and final annexation occurred under Claudius in 43 CE [85,86]. More than a century of heavy investment followed, which was financed by both the state and by local elites choosing to incorporate their lands into the Roman imperial system [87,88]. Monumental agorai, theatres, baths, aqueducts, and temples, were rapidly constructed in both coastal and upland Roman settlements (Figure 4b; [85,89]). Interconnectivity increased with infrastructural investment, and agricultural products critical for supply of Roman armies on the Danube and Syrian-Mesopotamian frontiers were more readily exported [90]. Roads were systematised, as evidenced by milestone inscriptions and the important Stadiasmus Patarensis inscription of regional road networks [91]. Lycian harbours became important nodes in regional grain markets and saw significant infrastructure upgrades. For example, granaries were built at Andriake and Patara, with a lighthouse at the latter [92]. Tax collection from cities was standardised across Roman territory and the state now profited from the rich resources of Lycia-Pamphylia, including traditional agricultural produce but also sponges, goat hair for ropes, wild animals for the circus, fish processed as garum, and, most importantly, timber [85,86]. Agricultural efficiency for the Mediterranean triad (grapes, grains

and olives) was enhanced with Roman innovation of the screw press for grapes and olives and the rotary mill for grain [93,94]. Roman hegemony also brought newfound security from unrest and a regional threat of banditry and looters [86], and might be reflected by a reduction in both urban fortifications and tower-farmsteads, as noted earlier [95,96]. All the above factors increased the importance of Lycia-Pamphylia for trade and enabled intensification of settlement and agriculture in the hinterlands [92,97]. These developments occurred despite prevailing climatic conditions that likely made agriculture more challenging when compared to the preceding and succeeding periods. The dense settlement detected in the Roman period continues into the Early Byzantine period, however a shift in the character and location of settlement is observed.

Late Antique prosperity and adaptation?

Settlement numbers peaked in the Roman period 50 BCE-350 CE, with the majority continuing into the Early Byzantine period 350-600 CE (189/276 Roman settlements: 68%). Overall settlement numbers remained high, with a slight decline in absolute numbers, with 255 of 381 settlements total (67%) dated to the Early Byzantine period. When bias from period length is counteracted by a time-adjustment (see Data Critique), the Early Byzantine period experiences the settlement evidence peak (Figure 2b). Important, however, is the shifting character and location of settlement between these two periods. There is a loss of settlement density in interior Lycia (Figures 3 and S3) caused by the abandonment of Roman Period highland rural settlements, which contributed to the highest mean elevation of abandonments for any period (814m asl). Dense settlement on the coast continues however, with 87 settlements <200m asl, including older Hellenistic and Roman cities (Figure S3). New

rural settlements also appear in the hinterlands of these cities, for instance around Fethiye, Olüdeniz, and Myra; the lowest mean elevation of new settlements for any period (514m asl) lowers the overall mean elevation (575m asl; Figures 3 and 4).

These findings are largely coherent with regional understanding. Though Martin Harrison [98] previously suggested that coastal cities in Lycia declined from the 5th century CE, with population and important settlements migrating inland to the mountainous regions, the inverse has otherwise been suggested – and is supported by our dataset – with increased settlement density in coastal regions and a possible decline in the interior regions [11,35,39,99,100]. The pollen data provides further support for this trend, with an Early Byzantine period decline in OJCV values at the high elevation Sögüt and Bereket Basin sites (Figure 7), coincident with the end of the BOP ended at these sites [15]. A decline in OJCV values is also observed in the other records from around Sagalassos (Ağlasun and Gravgaz Marsh), coincident with a reduction in settlement numbers in the hinterland survey (Figure 7; [78]). Similar to the Roman period, long-distance maritime transport of agricultural produce was important, with the state subsidising supply of food staples both to Constantinople and to Byzantine armies campaigning against Sassanid Persia [101,102]. This may have promoted expansion of settlements near to the coastlines and made inaccessible mountainside settlements comparatively less efficient [11,69]. Because lower coastal regions have enhanced precipitation compared to the higher elevation interior and have an extended growing season, they are more productive and allow for greater crop diversity [70]. The idea of a period of "prosperity" in the 4th-6th centuries CE is therefore more complicated, at least in Lycia-Pamphylia [11,35].

Adam Izdebski et al. [1] examined Early Byzantine "prosperity" in relation to climatic conditions, concluding that enhanced precipitation contributed to the expansion of rural settlement and agriculture into environmentally marginal terrain. However, at that time the only available high-resolution palaeoclimate records in Anatolia were those from Nar Gölü [103–105] and Sofular Cave [106], located in the Central Anatolian Plateau and Black Sea coastal regions, respectively. These records, and associated archaeological and historical data, suggest enhanced aridity 350-470 CE, followed by wetter conditions [1]. However, the climate of Lycia-Pamphylia is now better understood due to the local Kocain Cave record [19], which stresses spatial heterogeneity of climate in Anatolia. The record demonstrates that the SW region experienced the inverse pattern to that previously suggested, sharing more similarities with the Aegean [19,74]. Kocain $\delta^{18}O$ and Mg/Ca ratios indicate very high precipitation and effective moisture between 330 and 400-460 CE, followed by a rapid shift to drier conditions that persisted until ~830 CE (Figure 6). This is important as high-resolution variability of climatic conditions in SW Anatolia were previously unknown for most of this period. The Lake Salda record, for instance, starts at ~560 CE, and there are no historical references to climatic conditions from the region [20,21,107].

Reduced effective moisture from the mid-5th century CE may also be reflected in regional archaeological evidence for urban water infrastructure adaptations. Construction and refurbishment of large vaulted reservoirs for water storage escalated from the mid-5th century CE, with large-scale projects at seven of the regions major cities, including the well-excavated sites of Sagalassos, Patara, Rhodiapolis, Side, and Xanthos [108–111]. At Sagalassos, a reduction in the supply output of the older Roman aqueducts is visible in changes made to the city's fountains during the 6th century, with outlets cut at lower basin

levels in the Upper Agora, alongside construction of new metal and textile workshops. New public fountains were still constructed in Sagalassos; geochemical evidence from calcites in feeder channels indicates supply via rain-capture and snow-melt [108], contrary to traditional Roman patterns of urban water supply via spring-fed aqueducts [112,113]. New settlements entirely reliant on rainwater capture also appear in the Early Byzantine period, for instance at Lyrboton Kome [9] and Gemiler Adasi [114]. These urban water infrastructure adaptations coincide with the demonumentalisation and ruralisation of cities, the appearance of church architecture, and renewal of fortifications described below. These economising adaptations are traditionally associated in scholarship with "decline" but lately considered to be indicative of "adaptive reuse" [101], here possibly linked to drier climatic conditions revealed by the Kocain Cave multi-proxy record.

Early Byzantine settlement is most clearly evidenced by church architecture, which appears almost exclusively after the mid-5th century CE [9]. Intensified investment is indicated by the number of settlements evidenced by architecture (213/255 Early Byzantine settlements; Figure 2c), comprised mostly of church construction and renewal of fortifications. Roman *poleis* were required to have bishops, necessitating construction of churches and episcopal complexes. Church construction was frequently coincident with the demonumentalization and ruralization of city settlements. Churches were often well-integrated into Roman street plans but incorporated building materials from recently demolished Roman structures. They signalled more than change in religious belief, indicating new political and economic realities in cities [53,115]. Churches are also found in close proximity to agricultural developments in the countryside such as terracing (as at Asarcik, but unfortunately not dated; e.g., as in [116]) and in cities near artisanal installations (at Patara

and Sagalassos), or large scale water storage (at Xanthos and Gemiler Adasi). At Xanthos, for instance, recent excavations in the western agora have revealed two churches, one at the agora's center and one built into its western portico, surrounded by workshops and water installations that facilitated artisanal activity, all dated to the fifth and sixth centuries (Figure 4e; [117]). Whilst new churches are not necessarily indications of communal wealth, they did require significant investment [69,118]. This was especially true in Lycia where churches featured high-quality stone carving, even within village settings (e.g., north of Demre around Asarcık, Figure 4c; [119]). Lycian churches have long been a focus of scholarly attention (e.g., [46]), which, combined with their robustness, increases the archaeological visibility of rural settlements. These factors are perhaps reflected in the increase of rural settlements in our data between the Roman (43%: 119) and Early Byzantine (51%: 130) periods.

From the mid-5th century CE, older Roman cities saw growing industrialization throughout our region but began to lose their monumental aspect, alongside adaptation or reconstruction of older Roman public buildings (including theaters, baths, and agorai) for artisanal and industrial activities. For instance at Side, glass workshops appeared in the theater and around an abandoned temple in the agora after the 5th century [120,121]. At Limyra, baths near the theater were abandoned in the later fifth century CE and became home to workshops for textiles, metal and/or glass, and bone-carving [122]. At Andriake, a workshop for the extraction of precious murex dye was built in the 6th century using spolia from an older church: remaining were massive piles of murex shells and more than 22,000 amphorae fragments of the sixth and seventh centuries thought to be related to murex production and transport there [123,124].

Whilst reduced effective moisture appears in southwest Anatolia after ~450 CE, climatic shifts will not be homogenous across the Eastern Mediterranean. A complex web of interconnected natural and human factors for societal change in the Byzantine periods is suggested elsewhere to have its origin in the mid-6th century CE. These include cooling and drying associated with volcanic-induced reductions in solar irradiation, earthquakes, and epidemics, and insecurity resulting from conflicts between the Byzantine and Sasanian Empires, the Umayyad Caliphate, and nomadic groups. These are discussed in the below section.

Middle Byzantine Abandonment and Renucleation?

The transition between our Early Byzantine-Middle Byzantine periods, from c. 550 to 650 or 700 CE, shows a significant shift in the intensity and character of settlement. Settlement density was reduced in the Middle Byzantine period, with a total of 135 settlements. Of the settlements with Early Byzantine period evidence, 134/255 (52%) had been abandoned and only 17 new settlements are evidenced. An even more pronounced reduction is settlement density is suggested when the length of the longer period is considered, with time-adjustments (see Data Critique; Figure 2b).

Additionally, settlements were no longer occupied at the scales of the preceding Roman and Early Byzantine periods: In larger urban settlements elsewhere, a *citte ad isole* pattern is frequently observed, whereby small clusters of agro-industrial activity or “islands” are visible within the skeleton of older Roman and Early Byzantine cities, which were otherwise uninhabited. For example, Myra contained three separate fortified enclosures: one at the acropolis [125], one at the Church of St Nicholas, which was rebuilt as a domed

basilica in the 8th century CE [126], and one at the city's harbour, Andriake [35]. Antalya, which became a centre for the Byzantine navy and administration alongside significant investment in new architecture and fortifications may be one exception, though 20th century development has prevented any archaeological conclusions [9]. Furthermore, over half (72/135) of the Middle Byzantine settlements are only evidenced by church contraction. Very small chapels, often <3x5m^2 and sometimes with datable frescoes, are erected inside older derelict structures, mostly much larger church complexes [34] (see Figure 4f). Whilst church contractions could be indicative of the persistence of Christian holy spaces, they also suggest insecurity, inability, or unwillingness to invest, and demographic decline.

Investment in new construction or restorations is evidenced at only 45 settlements, a figure lower even than the Iron Age. This was largely comprised of smaller settlements where older Iron Age or Hellenistic circuits were refurbished (e.g., Idebessos, Tlos), and village settlements with small chapels constructed within older fortifications (e.g., at Asar Dağı, Trysa). Despite indications of both abandonment and reduced settlement intensity throughout our study region, continued investment in some of the larger cities suggests renucleation. Major church construction (e.g., at Antalya, Myra, Patara) and resettlement of disused urban acropolises (e.g., at Perge, Tlos, Xanthos) was accompanied by increased administrative importance [127]. Byzantine lead seals, which were used to secure documents and indicate information about the author [128], demonstrate the importance of Antalya, Myra, Perge, and Tlos, as centres of Byzantine naval and fiscal administration in the Kibyrrhaeot theme [9,129] (Table S3).

Other regional surveys, relying primarily on ceramic evidence, show divergent patterns (Figure 7). In the Sagalassos hinterland survey, settlement numbers had already

declined for the period starting at ~450 CE, whereas in the Dereköy Highland sherd counts remained high in their period starting 700 CE [78,130,131]. In the Balboura survey, sherd count increases in the 7th century CE, after re-dating of Cypriot Red Slip Ware (CRSW) by Armstrong [132] – a local/regional production that satisfies basic household needs [133]. In both surveys, the majority of Middle Byzantine sherds are local [134–137]. This pattern, which suggests a reduction in inter-regional exchange, is observed across the region, at settlements such as Sagalassos, Limyra and Xanthos [77,101,138]. This reduction in inter-regional exchange was accompanied by a reconfiguration in some contexts, away from Constantinople and towards the southeast: 7th-9th century CE deposits from Sagalassos contained LR7 amphora with fish remains from Egypt, and deposits from Limyra contained wares from Cyprus, Egypt, and the Levant [139].

The transition from the Early to Middle Byzantine periods also marks a major shift in the agricultural history of our study region and the Eastern Mediterranean more broadly. Intensive crop cultivation slowed considerably, pastoralism grew in importance, and re-wilding of the landscape occurred with pine forest recolonisation [79,140,141]. At the pollen sites where the BOP had not already ended (Gölhisar, Ağlasun, Gravgaz), it did in the 7th-8th CE (summarised in [15]). This is reflected in the OJCV values (Figures 7 and S4), which are minimal during the Middle Byzantine period.

Climate may have some responsibility for these changes in historical society within our study region. The new Kocain Cave Mg/Ca record (Figure 6c; [19]) suggests that in Lycia-Pamphylia, a rapid shift away from high effective moisture occurred ~460 CE and conditions remained relatively dry until at least 830 CE, except for short-lived breaks from aridity at ~571-578, 672-676 and 778-784 CE. Especially dry conditions occurred around the start of

the 7th century CE, with a minimum of precipitation indicated by Kocain $\delta^{18}O$ at ~590 CE and high Salda Ca/Fe ratios between ~620 and 650 CE overlain on a 600-770 CE dry-phase [21]. Very dry conditions are suggested again around the start of the 9th century CE, with Kocain Mg/Ca and $\delta^{18}O$ ratios consistently high between 790 and 830 CE and the start of a shift to less negative Gölhisar $\delta^{18}O$ ratios [19,20]. The initial aridification precedes the 536 CE eruption that resulted in a 12–18-month dust-veil covering much of the northern hemisphere, chronicled by Byzantine, Syrian and Chinese sources [142–144]. In other regions, climatic deterioration is suggested to have followed cooling resulting from this eruption and two others (540 and 547 CE), reinforced by sea-ice feedbacks, leading to the Late Antique Little Ice Age ("LALIA": 536-660 CE). However, this is now thought to have lasted for a shorter period of time, "the 536-550 CE climate downturn" [71,145]. The Kocain Cave record, and other Eastern Mediterranean records, perhaps support the suggestion that the 536 CE dust-veil event occurred superimposed over a longer-lasting pattern of cooling/drying climate known as the Dark Ages Cold Period ("DACP": 450-800 CE; [19,146]). Records from elsewhere in Anatolia, in Lake Nar and Sofular Cave, show either wetter conditions or no indication of a climatic impact from the 536-550 CE downturn [103–106], further stressing regional and local climatic variability.

An extended dry period is suggested for Lycia-Pamphylia (~460-830 CE) that would have lowered potential agricultural productivity, potentially disenabling arboriculture at higher elevations, and may have contributed to socio-economic and settlement decline. However, it is important to consider that there were earlier instances of dry climatic conditions where the local population appears to be relatively unafflicted, or even to have prospered. For example, during exceptionally dry conditions in the middle of the Iron Age

(800 and 700 BCE), Greek colonisation started in our region and, during the RCO agriculture thrived despite relatively low effective moisture. Additionally, the shift to drier conditions occurred at least a century earlier than the reduction in settlement numbers and investment, with a shift away from agriculture and towards pastoralism, which can be broadly dated to somewhere between 550 and 650 CE. This suggests that either the duration of the climatic change, or its combination with other factors was more important. These are primarily (1) pathogenic, (2) seismic, and (3) defensive.

(1) The appearance of the Plague of Justinian after 541 CE and lasting into the 8th century CE, an early epidemic of the bubonic plague caused by the bacterium *Yersinia pestis*, was traditionally understood as a mass mortality event, especially during the 540s CE [60,147]. Its presence in Lycia-Pamphylia is securely evidenced, with a detailed and contemporary account of widespread death at Myra, probably in 542 CE, that interrupted agricultural commerce from the Lycian interior to the port-city [107,148], and aDNA of *Yersinia pestis* identified from human remains at Sagalassos [149]. However, the demographic impact of the pandemic has recently been questioned [150–152]. The historical account of Myra also indicates recovery of the rural landscape thereafter [107,147,148] and, with the exception of the single victim at Sagalassos, aDNA is lacking for now [149]. Late Antique plague may have been a greater stimulus for shifts in religio-cultural beliefs and traditions than demographic or economic problems [153–155]. However, regional evidence indicates plague’s presence, and it cannot be excluded as a factor in the widespread settlement abandonment, and possible demographic contraction, that is clearly visible in the archaeology of Lycia-Pamphylia between the mid-6th and 7th centuries CE.

(2) Another major factor in 6th century changes elsewhere, and with evidence for in our study region, are earthquakes [73,156]. The Aegean tectonic plate, a subduction zone, nearly parallel to and offshore from the south Anatolian coast [157], causes frequent earthquakes in Lycia-Pamphylia [158]. An early 6th century (possibly 500 or 518 CE) earthquake occurred north of Antalya [159,160], collapsing numerous light buildings and the monumental gates at Sagalassos [161,162], with rebuilding activities widely visible in the city's archaeology [163]. In 528/9 CE, an earthquake centred on Fethiye-Meis [164] caused extensive damage across Lycia, particularly at Myra [165], with support given from imperial funds for reconstruction, according to typical protocols [156]. Communities suffering from earthquakes after the 6th century did not receive help or repair, however. Another earthquake around 600 CE "virtually wiped out Sagalassos as an urban settlement" [163] and further earthquakes in the seventh and eighth centuries have been observed in damaged and unrepaired structures at Side and around Myra [9].

(3) Regional insecurity also increased from the 7th century. Towards the end of the Byzantine-Sasanian conflicts, the Sasanians captured nearby Tarsus in 613 CE and Rhodes from 622/623 CE. The Umayyad caliphate's capture of Egypt in 642 CE ended the Annona grain supply to Constantinople, whose transport relied on Lycian harbour cities. An Umayyad navy formed in 649 CE that created coastal insecurity for centuries. Byzantine naval hegemony ended with defeat at the Battle of the Masts in 655, and was followed by direct raids on our study region in 666, 671-673, 676-678, 715, 807 and 1034 CE [166] (Table S3). As Douglas Baird [167] observes for the Konya Plain, investment in land for agriculture would quickly have become unstable upon the mere threat of seasonal Umayyad raids, which could have disrupted the economy and population without the need for destruction of property.

An interesting suggestion, beyond the scope of this paper, is that the complex web of interconnected factors (including climatic deterioration), which impacted varied regions across the northern hemisphere, may have "pushed" or "enabled" invasions by the Rashidun/Umayyad Caliphates [73], and nomadic groups [71].

Overall, evidence from Lycia-Pamphylia indicates settlement abandonment and contraction suggestive of demographic contraction, a shift from BOP agriculture to pastoralism, and the presence of dry climatic conditions, plague, earthquakes, and insecurity – all present at the transition between our Early and Middle Byzantine periods ("end of Late Antiquity") after the 6th century CE. However, clear causal attribution between archaeologically-visible developments and the aforementioned factors remain difficult to determine throughout Anatolia [168]. Evidence is overall stronger for the natural hazards, when compared to the evidence for societal response to them. Better chronological precision for archaeological change and site-specific studies of proxies for societal response (e.g., urban trash mounds [4]) are required to disentangle the causes of this change. Additionally, as with climate, there are earlier instances of plagues, earthquakes, and conflicts, where the population of Lycia-Pamphylia appear to have been unafflicted (see [156,169] for earthquakes). This emphasises these factors were contributory, rather than ultimate, and that it is ultimately socio-political factors that determine regional resilience and ability to adapt.

Conclusions

The regional record of historical settlements provided by the TIB 8, combined with regional climatic and environmental proxies, provides a clear picture of settlement change

for a region with previously disjointed evidence. Settlement numbers peak between the first century BCE and the sixth century CE in the Roman and Early Byzantine periods, alongside evolutions in the character and locations of settlement that begin ~460 CE, followed by a clear decline in absolute numbers that continues into the Middle Byzantine period. These shifts occur prior to the LALIA, increased seismic activity and the beginning of the Justinianic Plague, though they appear to have been intensified by the latter.

We find that, during the "Pax Romana" (1st and 2nd centuries CE), increasing agricultural productivity, settlement numbers, and population, with economic investment, does occur. However, linking these developments to warmer and wetter climatic conditions (as in the Roman Climatic Optimum [60]) is not possible in our study region, where conditions were drier than in the preceding and succeeding periods. During a previously proposed period of enhanced winter precipitation in the 4th-6th centuries CE [1], Lycia-Pamphylia experienced both enhanced effective moisture between 330 and 460 CE and dry conditions from 460 CE onward. This shift was concurrent with a change in the character of settlement, with urban water infrastructure possibly changing as an adaptation to drier conditions, demonumentalisation and ruralisation of cities that became agro-industrial villages, the appearance of church architecture, and renewal of fortifications. Drier conditions continued until at least 830 CE, and other factors with their origins in the mid-6th century CE, including recurrent plague epidemics, earthquakes and interstate conflict contributed to a significant decline in settlement numbers. Altogether, the individual contribution of each factor is hard to quantify and the combination of many factors into a "perfect storm" appears to be more important. The greatly reduced number of surviving Middle Byzantine settlements exhibit contraction of church complexes, occupied areas, and investment,

suggestive of population decline or changing societal priorities, though renucleation also occurs in some large centres.

Overall, we demonstrate that simple correlations between favourable (wetter) or adverse (drier) climatic conditions with positive or negative socio-economic conditions have numerous caveats, especially considering the spatial heterogeneity of both climatic and societal change in the Eastern Mediterranean. Our study region flourished during the drier Roman period, evolved during another period of aridity after 460 CE, but suffered under the weight of multiple pressures (political, environmental-climatic, seismic, pathogenic) after the mid-6th century CE.

Examining human-climate-environment interactions has been demonstrated as requiring strong archaeological and historical evidence, as well as high-quality palaeo-environmental and -climatic datasets. Further similar work can be done utilising earlier periods in this archaeological dataset. For example, interesting settlement, environmental, and climatic, changes are identified in the Iron Age and Hellenistic period. Additionally, similar archaeological datasets can be produced in existing and upcoming TIB regions [170], many of which contain highly-resolved speleothem and lake palaeoclimate records suitable for similar analysis: Cappadocia (Nar Lake [103]), Nicopolis and Cephalonia (Lake Trichonida [171]), Thrace (Uzuntarla Cave [172]), Bithynia (Sofular Cave [106,173]) and the Northern Aegean (Skala Marion Cave [174]). Furthermore, the Kocain Cave Mg/Ca record (and other high-resolution effective moisture records) may enable agroecosystem modelling of agricultural productivity for these regions (as in [37,175]).

Acknowledgements: to be completed later

Supplementary

Figures:

1. Resolution pie charts
2. Number of periods evidenced
3. Settlement change heatmaps
4. Full OJCV data

Tables:

1. Settlement dataset
2. Palaeo-data
3. Additional Byzantine evidence

Bibliography

1. Izdebski A, Pickett J, Roberts N, Waliszewski T. The environmental, archaeological and historical evidence for regional climatic changes and their societal impacts in the Eastern Mediterranean in Late Antiquity. Quat Sci Rev. 2016;136: 189–208. doi:10.1016/j.quascirev.2015.07.022
2. Roberts N, Cassis M, Doonan O, Eastwood W, Elton H, Haldon J, et al. Not the End of the World? Post-Classical Decline and Recovery in Rural Anatolia. Hum Ecol. 2018;46: 305–322. doi:10.1007/s10745-018-9973-2
3. Haldon J, Roberts N, Izdebski A, Fleitmann D, McCormick M, Cassis M, et al. The climate and environment of Byzantine Anatolia: Integrating science, history, and archaeology. Journal of Interdisciplinary History. 2014. doi:10.1162/JINH_a_00682
4. Bar-Oz G, Weissbrod L, Erickson-Gini T, Tepper Y, Malkinson D, Benzaquen M, et al. Ancient trash mounds unravel urban collapse a century before the end of Byzantine hegemony in the southern Levant. Proc Natl Acad Sci U S A. 2019;116: 8239–8248. doi:10.1073/pnas.1900233116
5. Vaiglova P, Hartman G, Marom N, Ayalon A, Bar-Matthews M, Zilberman T, et al. Climate stability and societal decline on the margins of the Byzantine empire in the Negev Desert. Sci Reports 2020 101. 2020;10: 1–13. doi:10.1038/s41598-020-58360-5
6. Horden P, Purcell N. The Corrupting Sea: A Study of Mediterranean History. Oxford: Blackwell Publishing Ltd; 2000.
7. Ulbrich U, Lionello P, Belušić D, Jacobeit J, Knippertz P, Kuglitsch G, et al. Climate of the Mediterranean: Synoptic Patterns, Temperature, Precipitation, Winds and their Extremes. In: Lionello P, editor. The Climate of the Mediterranean Region: From the Past to the Future. Amsterdam: Elsevier; 2012. pp. 301–346.
8. Luterbacher J, Garcia-Herrera R, Akçer-Ön S, Allan R, Alvarez-Castro MC, Benito G, et al. A review of 2000 years of paleoclimatic evidence in the Mediterranean. In: Lionello P, editor. The Climate of the Mediterranean region: from the past to the future. Amsterdam: Elsevier; 2012. pp. 87–185.
9. Hellenkemper H, Hild F. Tabula Imperii Byzantini 8: Lykien und Pamphylien. Vienna: Austrian Academy of Sciences Press; 2004.
10. Külzer A. Ein historischer Atlas zum byzantinischen Reich: Anfänge und Entwicklung der Tabula Imperii Byzantini (TIB). In: Külzer A, Polloczek V, Popović M St., editors. Raum und Geschichte: der historische Atlas 'Tabula Imperii Byzantini (TIB)' an der Österreichischen Akademie der Wissenschaften (Studies in Historical Geography and Cultural Heritage 3). Vienna-Novi Sad: Akademska knjiga; 2020. pp. 11–30.
11. Terpoy K. Questioning Late Antique prosperity: The case of Lycia (southwest Turkey). Byzantine Mod Greek Stud. 2019;43: 1–23. doi:10.1017/byz.2018.22
12. Izdebski A. A rural world in transition: Asia Minor from Late Antiquity into the early Middle Ages. Berlin: Taubenschlag Foundation; 2013.
13. Bakker J, Paulissen E, Kaniewski D, Poblome J, De Laet V, Verstraeten G, et al. Climate, people, fire and vegetation: New insights into vegetation dynamics in the Eastern Mediterranean since the 1st century AD. Clim Past. 2013;9: 57–87. doi:10.5194/cp-9-57-2013
14. Woodbridge J, Roberts CN, Palmisano A, Bevan A, Shennan S, Fyfe R, et al. Pollen-inferred regional vegetation patterns and demographic change in Southern Anatolia

through the Holocene. Holocene. 2019;29: 728–741. doi:10.1177/0959683619826635
15. Roberts N. Revisiting the Beyşehir Occupation Phase: Land-Cover Change and the Rural Economy in the Eastern Mediterranean During the First Millennium AD. Late Antiq Archaeol. 2018;11: 53–68. doi:10.1163/22134522-12340052
16. Mercuri AM, Bandini Mazzanti M, Florenzano A, Montecchi MC, Rattighieri E. Olea, Juglans and Castanea: The OJC group as pollen evidence of the development of human-induced environments in the Italian peninsula. Quat Int. 2013;303: 24–42. doi:10.1016/J.QUAINT.2013.01.005
17. Nemani RR, Keeling CD, Hashimoto H, Jolly WM, Piper SC, Tucker CJ, et al. Climate-driven increases in global terrestrial net primary production from 1982 to 1999. Science (80-). 2003;300: 1560–1563. doi:10.1126/science.1082750
18. Jones MD, Abu- Jaber N, AlShdaifat A, Baird D, Cook BI, Cuthbert MO, et al. 20,000 years of societal vulnerability and adaptation to climate change in southwest Asia. Wiley Interdiscip Rev Water. 2019;6: e1330. doi:10.1002/wat2.1330
19. Jacobson MJ, Flohr P, Gascoigne A, Leng MJ, Sadekov A, Cheng H, et al. Heterogenous Late Holocene Climate in the Eastern Mediterranean—The Kocain Cave Record From SW Turkey. Geophys Res Lett. 2021;48: 1–13. doi:10.1029/2021gl094733
20. Eastwood WJ, Leng MJ, Roberts N, Davis B. Holocene climate change in the eastern Mediterranean region: A comparison of stable isotope and pollen data from Lake Gölhisar, southwest Turkey. J Quat Sci. 2007;22: 327–341. doi:10.1002/jqs.1062
21. Danladi IB, Akçer-Ön S. Solar forcing and climate variability during the past millennium as recorded in a high altitude lake: Lake Salda (SW Anatolia). Quat Int. 2018;486: 185–198. doi:10.1016/j.quaint.2017.08.068
22. Quézel P, Médail F. Ecologie et biogéographie des forêts du bassin méditerranéen. Paris: Elsevier; 2003.
23. Sadori L, Bertini A, Combourieu-Nebout N, Kouli K, Mariotti Lippi M, Roberts N, et al. Palynology and Mediterranean vegetation history. Flora Mediterr. 2013;23: 141–156. doi:10.7320/FlMedit23.141
24. Tekeli FO, Gokce AF. Endemic plants and wild Allium species in Turkey. Acta Horticulturae. International Society for Horticultural Science; 2016. pp. 327–332. doi:10.17660/ActaHortic.2016.1143.46
25. Öztürk MZ, Çetınkaya G, Aydin S. Köppen-Geiger İklim Sınıflandırmasına Göre Türkiye'nin İklim Tipleri - (Climate Types of Turkey According to Köppen-Geiger Climate Classification). Istanbul Univ J Geogr. 2017;35: 17–27. doi:10.26650/jgeog330955
26. Beck HE, Zimmermann NE, McVicar TR, Vergopolan N, Berg A, Wood EF. Present and future köppen-geiger climate classification maps at 1-km resolution. Sci Data. 2018;5. doi:10.1038/sdata.2018.214
27. Yılmaz E, Çiçek İ. Türkiye'nin detaylandırılmış Köppen-Geiger iklim bölgeleri (Detailed Köppen-Geiger climate regions of Turkey). J Hum Sci. 2018;15: 225–242. doi:10.14687/jhs.v15i1.5040
28. Türkeş M, Erlat E. Winter mean temperature variability in Turkey associated with the North Atlantic Oscillation. Meteorol Atmos Phys. 2009;105: 211–225. doi:10.1007/s00703-009-0046-3
29. Sariş F, Hannah DM, Eastwood WJ. Spatial variability of precipitation regimes over Turkey. Hydrol Sci J. 2010;55: 234–249. doi:10.1080/02626660903546142

30. Çiner A. Coastal Landforms and Landscapes of Turkey. In: Kuzucuoğlu C, Çiner A, Kazancı N, editors. Landscapes and Landforms of Turkey. New York: Springer; 2019. pp. 233–247. doi:10.1007/978-3-030-03515-0_9
31. Kazancı N, Roberts N. The Lake Basins of South-west Anatolia. In: Kuzucuoğlu C, Çiner A, Kazancı N, editors. Landscapes and Landforms of Turkey. New York: Springer; 2019. pp. 325–337. doi:10.1007/978-3-030-03515-0_15
32. Giray H. Turkish agriculture at a glance. J Food, Agric Environ. 2012;10: 292–295.
33. McNeill JR. The Mountains of the Mediterranean World. Cambridge: Cambridge University Press; 1992.
34. Erdoğan O, Ceylan B. Church in Church: The Reuse of Late Antique Basilicas in Lycia in the Middle Ages (in Turkish). Cedrus. 2019;7: 663–679.
35. Foss C. The Lycian Coast in the Byzantine Age. Dumbart Oaks Pap. 1994;48: 1. doi:10.2307/1291721
36. Kowalewski SA. Regional Settlement Pattern Studies. Source J Archaeol Res. 2008;16: 225–285. doi:10.1007/S10814-008-9020-8
37. Contreras DA, Hiriart E, Bondeau A, Kirman A, Guiot J, Bernard L, et al. Regional paleoclimates and local consequences: Integrating GIS analysis of diachronic settlement patterns and processbased agroecosystem modeling of potential agricultural productivity in Provence (France). PLoS One. 2018;13: e0207622. doi:10.1371/journal.pone.0207622
38. Palmisano A, Bevan A, Shennan S. Comparing archaeological proxies for long-term population patterns: An example from central Italy. J Archaeol Sci. 2017;87: 59–72. doi:10.1016/j.jas.2017.10.001
39. Cassis M, Doonan O, Elton H, Newhard J. Evaluating Archaeological Evidence for Demographics, Abandonment, and Recovery in Late Antique and Byzantine Anatolia. 2018 [cited 23 Jul 2021]. doi:10.1007/s10745-018-0003-1
40. Barton CM, Bernabeu J, Aura JE, Garcia O. Land-use dynamics and socioeconomic change: an example from the Polop Alto Valley. Am Antiq. 1999;64: 609–634.
41. Plog FT. Diachronic Anthropology. In: Redman CL, editor. Research and Theory in Current Archaeology. New York: John Wiley & Sons, Ltd; 1973. pp. 181–198.
42. Dewar RE. Incorporating Variation in Occupation Span into Settlement-Pattern Analysis. Am Antiq. 1991;56: 604–620.
43. Premo LS. Reports Cultural Transmission and Diversity in Time-Averaged Assemblages. Curr Anthropol. 2014;55. doi:10.1086/674873
44. Schacht RM. The Contemporaneity Problem. Am Antiq. 1984;49: 678–695.
45. Garvey R. Current and potential roles of archaeology in the development of cultural evolutionary theory. Philos Trans R Soc B Biol Sci. 2018;373. doi:10.1098/RSTB.2017.0057
46. Fellows C. An account of discoveries in Lycia, being a journal kept during a second excursion in Asia Minor. London: J. Murray; 1840.
47. Orton C, Hughes M. Pottery in Archaeology. 2nd Editio. Cambridge: Cambridge University Press; 2013.
48. Hirschon R. Crossing the Aegean: An Appraisal of the 1923 Compulsory Population Exchange Between Greece and Turkey. Oxford: Berghahn Books; 2003.
49. Cesaretti R, Lobo J, Bettencourt L, Ortman S, Smith ME. Population-Area Relationship for Medieval European Cities. PLoS One. 2016;11.

doi:https://doi.org/10.1371/journal.pone.0162678
50. Hansen MH. Polis: An Introduction to the Ancient Greek City-State. Oxford: Oxford University Press; 2006.
51. Grainger JD. The Cities of Pamphylia. Oxford: Oxbow Books; 2009.
52. Elton H. The countryside in southern Asia Minor in the long sixth century. In: Elton H, Jacobs I, editors. Asia Minor in the Long Sixth Century: Current Research and Future Directions. Oxford: Oxbow Books; 2019. pp. 91–108. doi:10.2307/j.ctvjsf69w
53. Saradi HG. The Byzantine City in the Sixth Century: Literary Images and Historical Reality. Athens: Society for Messenian Archaeological Studies; 2006.
54. Smith ME. The archaeological study of neighbourhoods and districts in ancient cities. J Anthropol Archaeol. 2010;29: 137–154. doi:https://doi.org/10.1016/j.jaa.2010.01.001
55. Alcock S, Cherry J. Side-by-Side Survey: Comparative Regional Studies in the Mediterranean World. Oxford: Oxbow Books; 2016.
56. Roberts N, Woodbridge J, Palmisano A, Bevan A, Fyfe R, Shennan S. Mediterranean landscape change during the Holocene: Synthesis, comparison and regional trends in population, land cover and climate. Holocene. 2019;29: 923–937. doi:10.1177/0959683619826697
57. Degroot D, Anchukaitis KJ, Bauch M, Burnham J, Carnegy F, Cui J, et al. Towards a rigorous understanding of societal responses to climate change. Nature. 2021;591: 539–550. doi:10.1038/s41586-021-03190-2
58. Ellenblum R. The Collapse of the Eastern Mediterranean: Climate Change and the Decline of the East, 950-1072. Cambridge: Cambridge University Press; 2012.
59. Cline EH. 1177 B.C.: The Year Civilization Collapsed. Princeton, New Jersey: Princeton University Press; 2014.
60. Harper K. The Fate of Rome. Princeton, New Jersey: Princeton University Press; 2017.
61. Knapp AB, Manning SW. Crisis in Context: The End of the Late Bronze Age in the Eastern Mediterranean. Am J Archaeol. 2016;120: 99–149. doi:10.3764/aja.120.1.0099
62. Contreras DA. The Archaeology of Human-Environment Interactions: Strategies for Investigating Anthropogenic Landscapes, Dynamic Environments, and Climate Change in the Human Past. Contreras DA, editor. New York: Routledge; 2016. doi:10.4324/9781315697697
63. Decker MJ. Approaches to the environmental history of Late Antiquity, part 1: The rise of Islam. Hist Compass. 2017;15: e12407. doi:10.1111/hic3.12407
64. Decker MJ. Approaches to the environmental history of Late Antiquity, part II: Climate Change and the End of the Roman Empire. Hist Compass. 2017;15: e12425. doi:10.1111/hic3.12425
65. Haldon J, Chase AF, Eastwood W, Medina-Elizalde M, Izdebski A, Ludlow F, et al. Demystifying Collapse: Climate, environment, and social agency in pre-modern societies. Millennium. 2020;17: 1–33. doi:10.1515/mill-2020-0002
66. Cornwell H. Pax and the Politics of Peace: Republic to Principate. Oxford: Oxford University Press; 2017.
67. Goldsworthy A. Pax Romana: War, Peace and Conquest in the Roman World. New Haven: Yale University Press; 2016.
68. Haldon J, Mordechai L, Newfield TP, Chase AF, Izdebski A, Guzowski P, et al. History meets palaeoscience: Consilience and collaboration in studying past societal responses to environmental change. Proc Natl Acad Sci U S A. 2018;115: 3210.

doi:10.1073/PNAS.1716912115
69. Izdebski A. The Economic Expansion of the Anatolian Countryside in Late Antiquity: The Coast Cersus Inland Regions. Local Economies?: Production and Exchange of Inland Regions in Late Antiquity. 2013. pp. 343–376. doi:10.1163/22134522-12340036
70. Decker MJ. Tilling the Hateful Earth: Agricultural Production and Trade in the Late Antique East. Oxford: Oxford University Press; 2009.
71. Büntgen U, Myglan VS, Ljungqvist FC, McCormick M, Di Cosmo N, Sigl M, et al. Cooling and societal change during the Late Antique Little Ice Age from 536 to around 660 AD. Nat Geosci. 2016;9: 231–236. doi:10.1038/ngeo2652
72. Helama S, Arppe L, Uusitalo J, Holopainen J, Mäkelä HM, Mäkinen H, et al. Volcanic dust veils from sixth century tree-ring isotopes linked to reduced irradiance, primary production and human health. Sci Rep. 2018;8: 1–12. doi:10.1038/s41598-018-19760-w
73. Korotayev A V., Klimenko V, Proussakov D. Origins of Islam: political-anthropological and environmental context. Acta Orient Acad Sci Hungaricae. 1999;52: 243–276.
74. Labuhn I, Finné M, Izdebski A, Roberts N, Woodbridge J. Climatic Changes and Their Impacts in the Mediterranean during the First Millennium AD. Late Antiq Archaeol. 2018;12: 65–88. doi:10.1163/22134522-12340067
75. Morris S. Greek Towers and Slaves: An Archaeology of Exploitation. J Archaeol. 2005;109: 155–225.
76. Waelkens M. Sagalassos V. Report on the Survey and Excavation Campaigns of 1996 and 1997. Leuven: Leuven University Press; 2000.
77. Poblome J, Talloen P, Kaptijn E. Sagalassos. In: Niewöhner P, editor. The Archaeology of Byzantine Anatolia: From the End of Late Antiquity until the Coming of the Turks. Oxford: Oxford University Press; 2017. pp. 302–311.
78. Vandam R, Kaptijn E, Willet R, Willett PT. The Countryside - Where Are The People? In: Poblome J, Torun E, Talloen P, Waelkens M, editors. Meanwhile in the Mountains: Sagalassos. Istanbul: Yapı Kredi Yayınları; 2019. pp. 275–284.
79. De Cupere B, Frémondeau D, Kaptijn E, Marinova E, Poblome J, Vandam R, et al. Subsistence economy and land use strategies in the Burdur province (SW Anatolia) from prehistory to the Byzantine period. Quat Int. 2017;436: 4–17. doi:10.1016/j.quaint.2015.11.097
80. Bakker J, Kaniewski D, Verstraeten G, De Laet V, Waelkens M. Numerically derived evidence for late-Holocene climate change and its impact on human presence in the southest Taurus Mountains, Turkey. Holocene. 2011;22: 425–438. doi:10.1177/0959683611425546
81. Koç K, Koşun E, Cheng H, Demirtaş F, Lawrence Edwards R, Fleitmann D. Black carbon traces of human activities in stalagmites from Turkey. J Archaeol Sci. 2020;123: 105255. doi:10.1016/J.JAS.2020.105255
82. Haldon J, Elton H, Huebner SR, Izdebski A, Mordechai L, Newfield TP. Plagues, climate change, and the end of an empire: A response to Kyle Harper's The Fate of Rome (1): Climate. Hist Compass. 2018;16: e12508. doi:10.1111/hic3.12508
83. Heinrich I, Touchan R, Dorado Liñán I, Vos H, Helle G. Winter-to-spring temperature dynamics in Turkey derived from tree rings since AD 1125. Clim Dyn. 2013;41: 1685–1701. doi:10.1007/s00382-013-1702-3
84. Mitchell S. The Treaty between Rome and Lycia of 46 BC (MS 2070). In: Pintaudi R,

editor. Papyri Graecae Schøyen The Schøyen Collection V: Greek Papyri Vol I. Florence: Gonnelli; 2005. pp. 165–258.
85. Brandt H, Kolb F. Lycia et Pamphylia: eine römische Provinz im südwesten Kleinasiens. Mainz: Verlag Philipp von Zabern; 2005.
86. Bennett J. Why did Claudius Annex Lycia? Adalya. 2011;14: 119–136.
87. Kokkinia C. Die Opramoas-Inschrift von Rhodiapolis: Euergetismus und Soziale Elite in Lykien. Bonn: Rudolf Habelt; 2000.
88. Waelkens M. Romanization in the East. A Case Study: Sagalasso and Pisidia. Istanbuler Mitteilungen. 2002;52: 311–368.
89. Milner NP. Building Roman Lycia: new inscriptions and monuments from the baths and peristyle buildings Ml 1 and Ml 2 at Oinoanda. Anatol Stud. 2016;66: 91–124.
90. Akyürek E. Andriake: The Port of Myra in Late Antiquity. In: Magdalino P, Necipoğlu N, editors. Trade in Byzantium: Papers from the Third International Sevgi Gönül Byzantine Studies Symposium. Istanbul: Köc University Research Center for Anatolian Civilizations; 2016. pp. 465–487.
91. Adak M, Şahin S. Stadiasmus Patarensis: Itinera Romana Provinciae Lyciae. Istanbul: Ege Yayınları; 2007.
92. Rice C. The ports of Roman Lycia: Urbanism, networks, and hierarchies. In: Flohrr ME, editor. Urban Space and Urban History in the Roman World. New York: Routledge; 2020. pp. 241–265.
93. Curtis R. Food Processing and Preparation. In: Oleson JP, editor. Oxford Handbook of Engineering and Technology in the Classical World. Oxford: Oxford University Press; 2008. pp. 369–392.
94. Greene K. Technological Innovation and Economic Progress in the Ancient World: M. I. Finley Re-Considered. Econ Hist Rev. 2000;53: 29–59.
95. Çevik N, Bulut S. The Belen and Kelbessos farmsteads with towers on the border of Pisidia-Lycia and some thoughts on security in the countryside. Adalya. 2007;10: 105–130.
96. Takaoğlu T. Adapting to a Diverse Landscape: Agriculture in Hellenistic and Roman Anatolia. In: Hollander DB, Howe T, editors. A Companion to Ancient Agriculture. Hoboken, New Jersey: John Wiley & Sons, Ltd; 2020. pp. 363–382. doi:10.1002/9781118970959.ch17
97. Dündar E. The Maritime of Roman Patara. Preliminary Remarks on the Amphorae. Skyliss. 2018;2: 166–173.
98. Harrison M. Mountain and Plain. Ann Arbor: University of Michigan Press; 2001.
99. Foss C. Lycia in history. In: Morganstern J, editor. The Fort at Dereağzı and Other Material Remains in its Vicinity: From Antiquity to the Middle Ages. Tubingen: Ernst Wasmuth Verlag; 1993. pp. 5–25.
100. Terpoy K. Studying Asia Minor in the sixth century. Methodological considerations for an economic analysis. In: Elton H, Jacobs I, editors. Asia Minor in the Long Sixth Century: Current Research and Future Directions. Oxford: Oxbow Books; 2019. pp. 63–78. doi:https://doi.org/10.2307/j.ctvjsf69w
101. Commito A. The cities of southern Asia Minor in the sixth century. Asia Minor Long Sixth Century. 2019; 109–142. doi:10.2307/j.ctvjsf69w.10
102. Morrisson C, Sodini J-P. The Sixth-Century Economy. In: Laiou AE, editor. The Economic History of Byzantium. Washington, D.C.: Dumbarton Oaks; 2002. pp. 171–

220.
103. Dean JR, Jones MD, Leng MJ, Metcalfe SE, Sloane HJ, Eastwood WJ, et al. Seasonality of Holocene hydroclimate in the Eastern Mediterranean reconstructed using the oxygen isotope composition of carbonates and diatoms from Lake Nar, central Turkey. Holocene. 2018;28: 267–276. doi:10.1177/0959683617721326
104. Dean JR, Jones MD, Leng MJ, Noble SR, Metcalfe SE, Sloane HJ, et al. Eastern Mediterranean hydroclimate over the late glacial and Holocene, reconstructed from the sediments of Nar lake, central Turkey, using stable isotopes and carbonate mineralogy. Quat Sci Rev. 2015;124: 162–174. doi:10.1016/j.quascirev.2015.07.023
105. Jones MD, Roberts N, Leng MJ, Türkeş M. A high-resolution late Holocene lake isotope record from Turkey and links to North Atlantic and monsoon climate. Geology. 2006;34: 361–364. doi:10.1130/G22407.1
106. Göktürk OM, Fleitmann D, Badertscher S, Cheng H, Edwards RL, Leuenberger M, et al. Climate on the southern Black Sea coast during the Holocene: Implications from the Sofular Cave record. Quat Sci Rev. 2011;30: 2433–2445. doi:10.1016/j.quascirev.2011.05.007
107. McCormick M, Büntgen U, Cane MA, Cook ER, Harper K, Huybers P, et al. Climate Change during and after the Roman Empire: Reconstructing the Past from Scientific and Historical Evidence. J Interdiscip Hist. 2012;43: 169–220. doi:10.1162/JINH_a_00379
108. Martens F. Water Abundance and Shortage at Sagalassos (SW-Turkey). In: Ohlig C, editor. Cura Aquarum in Jordanien Beiträge des 13 Internationalen Symposiums zur Geschichte der Wasserwirtschaft und des Wasserbaus im Mediterranen Raum Petra/Amman 31 März – 09 April 2007. Siegburg: DWhG; 2008. p. 247=262.
109. Işkan H. Neue Ergebnisse zur Wasserleitung von Patara / Türkei. In: Wiplinger G, editor. Historische Wasserleitungen Gestern – Heute – Morgen Tagungsband des internationalen Frontinus-Symposiums, Wien 19 – 23 Oktober 2011. Leuven: Peeters; 2013. pp. 93–103.
110. Akyürek E, Tiryaki A. Rhodiapolis Piskoposluk Kilisesi Kazılarından Üç Mimari Plastik Eser Üzerine Değerlendirmeler. Adalya. 2010;13: 398–403.
111. Sodini J-P, Canbilen H, Lebouteiller P. La basilique de l'acropole haute de Xanthos. Anatolia Antiq. 1996;4: 201–229.
112. Pickett J. Water and empire in the de aedificiis of procopius. Dumbart Oaks Pap. 2017;71: 95–125.
113. Pickett J. Hydraulic Landscapes of Cities in the East Roman World. In: Beardsley J, Farhat G, editors. Landscapes of Pre-industrial Urbanism. Washington, D.C.: Dumbarton Oaks; 2020.
114. Asano K. Island of St. Nicholas. Excavation and Survey of the Gemiler Island Area, Lycia, Turkey. Osaka: Osaka University Press; 2010.
115. Haldon J. The Empire That Would Not Die: The Paradox of Eastern Roman Survival, 640–740. Cambridge: Harvard University Press; 2016. doi:10.1093/ehr/cex279
116. Turner S. Agricultural terraces in the Mediterranean: medieval intensification revealed by OSL profiling and dating. Antiquity. 2021;95: 773–790.
117. Dönmez A. Xanthos West Agora II: Alteration and Transformation in the Byzantine Period. Adalya. 2018;21: 277–314.
118. Bowden W. A new urban élite? Church builders and church building in late-antique

Epirus. In: Lavan L, Bowden W, editors. Recent research in late-antique urbanism. Portsmouth: Journal of Roman Archaeology; 2001. pp. 57–67.
119. Grossmann P, Severin H-G. Frühchristliche und byzantinische Bauten im südöstlichen Lykien: Ergebnisse zweier Surveys. Istanbuler Forschungen Bd. 46. Tübingen: Ernst Wasmuth Verlag; 2003.
120. Alanyalı HS. Work at and around Side Theater in 2009. ANMED. 2010;8: 94–103.
121. Alanyalı HS. Excavations at Side in 2010. ANMED. 2011;9: 100–112.
122. Österreichischen Archäologischen Instituts. Wissenschaftlicher Jahresbericht des Österreichischen Archäologischen Instituts. Vienna: ÖAW; 2017.
123. Yener-Marksteiner B. Surveys in Andriake 2008. ANMED. 2009;7: 105–107.
124. Çevik N, Bulut S. Excavations at Myra and Andriake in 2010. ANMED. 2011;9: 59–70.
125. Borchhardt J. Myra: eine Lykische Metropole in antiker und byzantinischer Zeit. Gebr. Berlin: Mann Verlag; 1975.
126. Ousterhout R. Eastern Medieval Architecture. The Building Traditions of Byzantium and Neighboring Lands. Oxford: Oxford University Press; 2019.
127. Peschlow U. Patara. In: Niewöhner P, editor. The Archaeology of Byzantine Anatolia: From the End of Late Antiquity until the Coming of the Turks. Oxford: Oxford University Press; 2017. pp. 280–291.
128. Oikonomides N. The Usual Lead Seal. Dumbart Oaks Pap. 1983;37: 147–157.
129. Brubaker L, Haldon J. Byzantium in the iconoclast era c. 680-850: a history. Cambridge: Cambridge University Press; 2011.
130. Vandam R, Kaptijn E, Broothaerts N, De Cupere B, Marinova E, Van Loo M, et al. "Marginal" Landscapes: Human Activity, Vulnerability, and Resilience in the Western Taurus Mountains (Southwest Turkey). J East Mediterr Archaeol Herit Stud. 2019;7: 432–450. doi:doi: 10.5325/jeasmedarcherstu.7.4.0432
131. Vandam R, Willett PT, Poblome J. The Results of the 2017 Dereköy Archaeological Survey by the Sagalassos Project in the Western Taurus Mountains. Archaeol Anatolia Vol III Recent Discov. 2019; 260–269.
132. Armstrong P. Trade in the east Mediterranean in the 8th century. In: Mango MM, editor. Byzantine Trade, 4th-12th Centuries. Aldershot: Ashgate Publishing; 2009. pp. 157–178.
133. Jackson M, Zelle M, Vandeput L, Köse V. Primary evidence for Late Roman D Ware production in southern Asia Minor: a challenge to 'Cypriot Red Slip Ware.' Anatol Stud. 2012;62: 89–114.
134. Coulton JJ. The Balboura Survey and Settlement in Highland Southwest Anatolia: Volume 2. The Balboura survey: detailed studies and catalogues. London: British Institute of Archaeology at Ankara; 2012.
135. Coulton JJ. The Balboura Survey and Settlement in Highland Southwest Anatolia: Volume 1. Balboura and the history of highland settlement. London: British Institute of Archaeology at Ankara; 2012.
136. Armstrong P. The survey pottery: Hellenistic and later. In: Coulton JJ, editor. The Balboura Survey and Settlement in Highland Southwest Anatolia: Volume 2 The Balboura survey: detailed studies and catalogues. London: British Institute of Archaeology at Ankara; 2012. pp. 31–82.
137. Vionis AK, Poblome J, Waelkens M. The hidden material culture of the Dark Ages. Early medieval ceramics at Sagalassos (Turkey): new evidence (ca AD 650–800). Anatol Stud.

2009;59: 147–165. doi:10.1017/S0066154600000946
138. Cavalier L. Observations sur l'architecture de Xanthos a l'époque archaïque. Rev des Etudes Anciennes. 2006;108: 327–354.
139. Vroom J. The Other Dark Ages: Early Medieval Pottery Finds in the Aegean as an Archaeological Challenge. In: Attoui R, editor. When did Antiquity End? Archaeological Case Studies in Three Continents. Oxford: Archaeopress; 2011. pp. 137–158.
140. Izdebski A. Why did agriculture flourish in the late antique East? The role of climate fluctuations in the development and contraction of agriculture in Asia Minor and the Middle East from the 4th till the 7th c. AD. Millennium. 2011;8: 291–312. doi:10.1515/9783110236453.291
141. Eastwood WJ, Roberts N, Lamb HF. Palaeoecological and archaeological evidence for human occupance in southwest Turkey: the Beyşehir occupation phase. Anatol Stud. 1998;48: 69–86. doi:10.2307/3643048
142. Arjava A. The Mystery Cloud of 536 CE in the Mediterranean Sources. Dumbart Oaks Pap. 2005;59: 73–94.
143. Houston MS. Chinese Climate, History, and State Stability in AD 536. In: Gunn JD, editor. The Years without Summer: Tracing AD 536 and Its Aftermath. Oxford: BAR International Series; 2000. pp. 71–77.
144. Stothers RB. Cloudy and clear stratospheres before A.D. 1000 inferred from written sources. J Geophys Res Atmos. 2002;107. doi:10.1029/2002JD002105
145. Newfield TP. The Climate Downturn of 536-50. In: Whites S, Pfister C, Mauelshagen F, editors. The Palgrave Handbook of Climate History. London: Palgrave Macmillan; 2018. pp. 447–493. doi:10.1057/978-1-137-43020-5
146. Helama S, Jones PD, Briffa KR. Dark Ages Cold Period: A literature review and directions for future research. Holocene. 2017;27: 1600–1606. doi:10.1177/0959683617693898
147. Stathakopoulos DC. Famine and Pestilence in the Late Roman and Early Byzantine Empire: A Systematic Survey of Subsistence Crises and Epidemics (Birmingham Byzantine and Ottoman Studies). Aldershot: Ashgate Publishing; 2004.
148. Ševčenko I, Ševčenko NP. The Life of Saint Nicholas of Sion. Text and Translation (Archbishop Iakovos Library of Ecclesiastical and Historical Sources 10). Brookline, Mass.: Hellenic Collage Press; 1984.
149. Van de Vijver K. Fiziksel antropoloji çalışmaları. 39 Kazı sonuçları toplantısı 22–26 Mayış 2017 Bursa. Ankara: T.C. Kültür ve Turizm Bakanlığı; 2018. pp. 227–228.
150. White LA, Mordechai L. Modeling the Justinianic Plague: Comparing hypothesized transmission routes. PLoS One. 2020;15. doi:10.1371/journal.pone.0231256
151. Eisenberg M, Mordechai L. The Justinianic Plague: An interdisciplinary review. Byzantine and Modern Greek Studies. Cambridge University Press; 2019. pp. 156–180. doi:10.1017/byz.2019.10
152. Mordechai L, Eisenberg M, Newfield TP, Izdebski A, Kay JE, Poinar H. The Justinianic Plague: An inconsequential pandemic? Proc Natl Acad Sci U S A. 2019;116: 25546–25554. doi:10.1073/pnas.1903797116
153. Meier M. The 'Justinianic Plague': An "Inconsequential Pandemic"? A Reply. Medizinhist J. 2020;55: 172–199. doi:10.25162/MHJ-2020-0006
154. Meier M. Prokop, Agathias, die Pest und das 'Ende' der antiken Historiographie. Naturkatastrophen und Geschichtsschreibung in der ausgehenden Spätantike. Hist

Zeitschrif. 2004;278: 281–310.
155. Meier M. The 'Justinianic Plague'. The Economic Consequences of the Pandemic in the Eastern Roman Empire and Its Cultural and Religious Effects. Early Mediev Eur. 2016;24.
156. Mordechai L, Pickett J. Earthquakes as the Quintessential SCE: Methodology and Societal Resilience. Hum Ecol. 2018;46: 335–348. doi:10.1007/s10745-018-9985-y
157. Bird P. An updated digital model of plate boundaries. Geochemistry, Geophys Geosystems. 2003;4: 1027. doi:https://doi.org/10.1029/2001GC000252
158. Ambraseys N. Earthquakes in the eastern Mediterranean and the Middle East: a multidisciplinary study of 2000 years of seismicity. Cambridge: Cambridge University Press; 2009.
159. Ergin K, Guclu U, Uz Z. A Catalog of Earthquakes for Turkey and the Surrounding Area (11 A.D. to 1964 A.D.). Istanbul: Istanbul Technical Univeristy; 1967.
160. Similox-Tohon D, Sintubin M, Muchez P, Verhaert G, Vanneste K, Fernandez M, et al. The identification of an active fault by a multidisciplinary study at the archaeological site of Sagalassos (SW Turkey). Tectonophysics. 2006;420: 371–387. doi:10.1016/j.tecto.2006.03.026
161. Gates M-H. Archaeology in Turkey. Am J Archaeol. 1997;101: 241. doi:10.2307/506511
162. Waelkens M, Sintubin M, Muchez P, Paulissen E. Archaeological, geomorphological and geological evidence for a major earthquake at Sagalassos (SW Turkey) around the middle of the seventh century AD. Geol Soc Spec Publ. 2000;171: 373–383. doi:10.1144/GSL.SP.2000.171.01.27
163. Waelkens M. Sagalassos, Archaeology of. 2nd editio. In: Smith C, editor. Encyclopedia of Global Archaeology. 2nd editio. Basel: Springer; 2019. pp. 1121–1123.
164. Erel T, Adatepe F. Traces of historical earthquakes in the ancient city life at the Mediterranean region. J Black Sea / Mediterr Environ. 2007;13: 241–252.
165. Malalas J. The Chronicle of John Malalas. Jeffreys E, Jeffreys M, Scott R, editors. The Chronicle of John Malalas. Melbourne: Australian Association for Byzantine Studies; 2017. doi:10.1163/9789004344600
166. Mango C, Scott R, Greatrex G. The Chronicle of Theophanes Confessor: Byzantine and Near Eastern History, AD 284-813. Oxford: Oxford University Press; 1997.
167. Baird D. Settlement expansion on the Konya Plain, Anatolia 5th-7th centuries AD. In: Lavan L, Bowden W, editors. The Late Antique Countryside. Oxford: Brill; 2004. pp. 219–246.
168. Mitchell S. The Great Plague of Late Antiquity in Asia Minor. In: Şimşek C, Kaçar T, editors. The Lykos Valley and Neighborhood in Late Antiquity. Istanbul: Ege Yayınları; 2018. pp. 27–36.
169. Mordechai L. Short-Term Cataclysmic Events in Premodern Complex Societies. Hum Ecol. 2018;46: 323–333.
170. Külzer A. Reconstructing medieval landscapes: the Austrian research project Tabula Imperii Byzantini and its work in Western Anatolia. In: Prevosti M, Duran JGI, editors. Proceedings of the 1st Tir-For Symposium From territory studies to digital cartography. Barcelona: Biblioteca de Catalunya; 2021. pp. 91–102. doi:10.2436/15.1000.02.8
171. Seguin J, Avramidis P, Dörfler W, Emmanouilidis A, Unkel I. A 2600-year high-resolution climate record from Lake Trichonida (SW Greece). E&G Quat Sci J.

2020;69: 139–160. doi:10.5194/egqsj-69-139-2020
172. Göktürk OM. Climate in the Eastern Mediterranean through the Holocene inferred from Turkish stalagmites (PhD Thesis). University of Bern, Switzerland. 2011.
173. Fleitmann D, Cheng H, Badertscher S, Edwards RL, Mudelsee M, Göktürk OM, et al. Timing and climatic impact of Greenland interstadials recorded in stalagmites from northern Turkey. Geophys Res Lett. 2009;36: 1–5. doi:10.1029/2009GL040050
174. Psomiadis D, Dotsika E, Albanakis K, Ghaleb B, Hillaire-Marcel C. Speleothem record of climatic changes in the northern Aegean region (Greece) from the Bronze Age to the collapse of the Roman Empire. Palaeogeogr Palaeoclimatol Palaeoecol. 2018;489: 272–283. doi:10.1016/j.palaeo.2017.10.021
175. Contreras DA, Bondeau A, Guiot J, Kirman A, Hiriart E, Bernard L, et al. From paleoclimate variables to prehistoric agriculture: Using a process-based agro-ecosystem model to simulate the impacts of Holocene climate change on potential agricultural productivity in Provence, France. Quat Int. 2019;501: 303–316. doi:10.1016/J.QUAINT.2018.02.019

Figure 1

Click here to access/download;Figure;FIG1MA~1.TIF

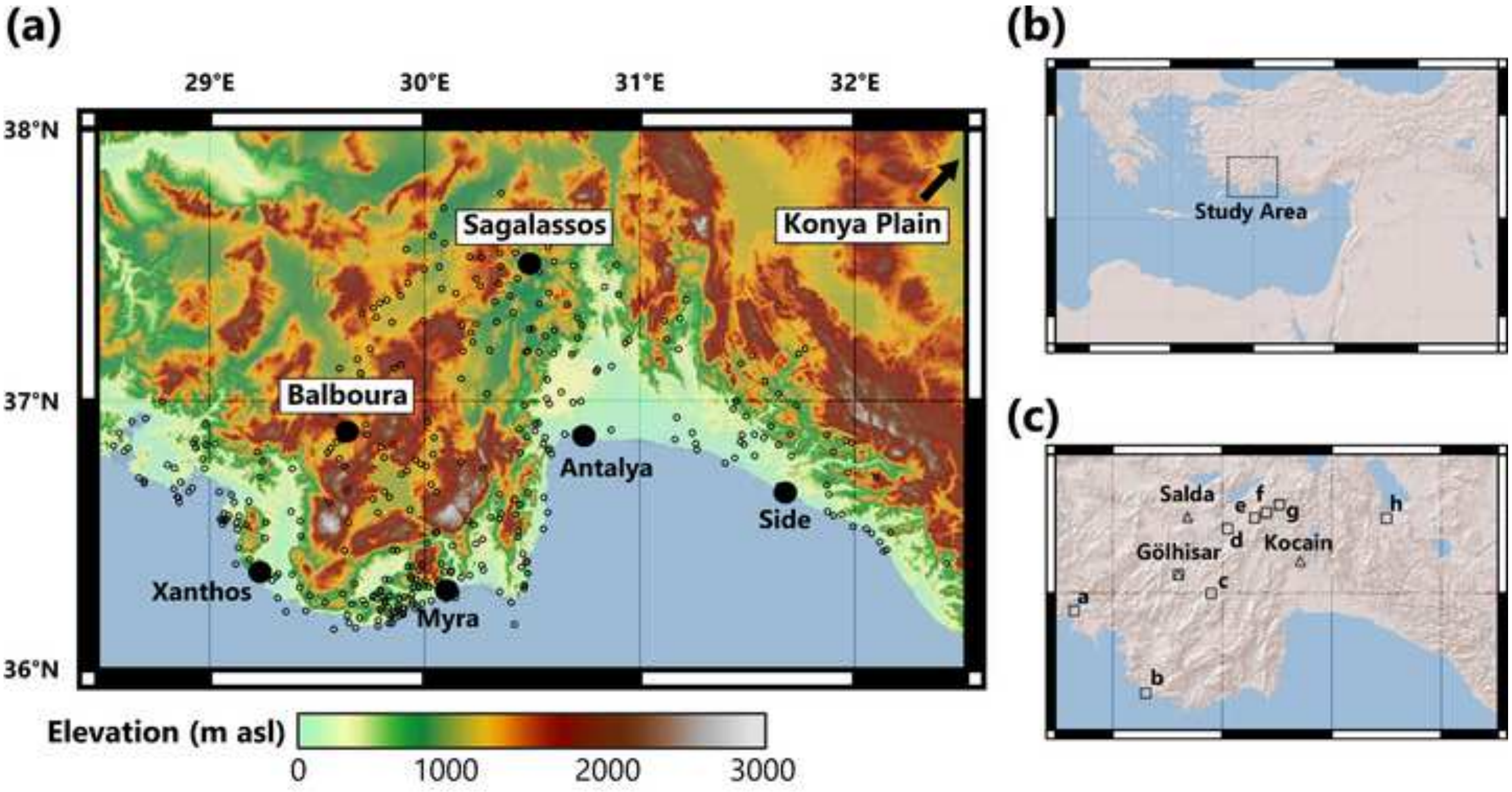

Figure 2

Click here to access/download;Figure;Fig 2 TIB data_TIFF.tif

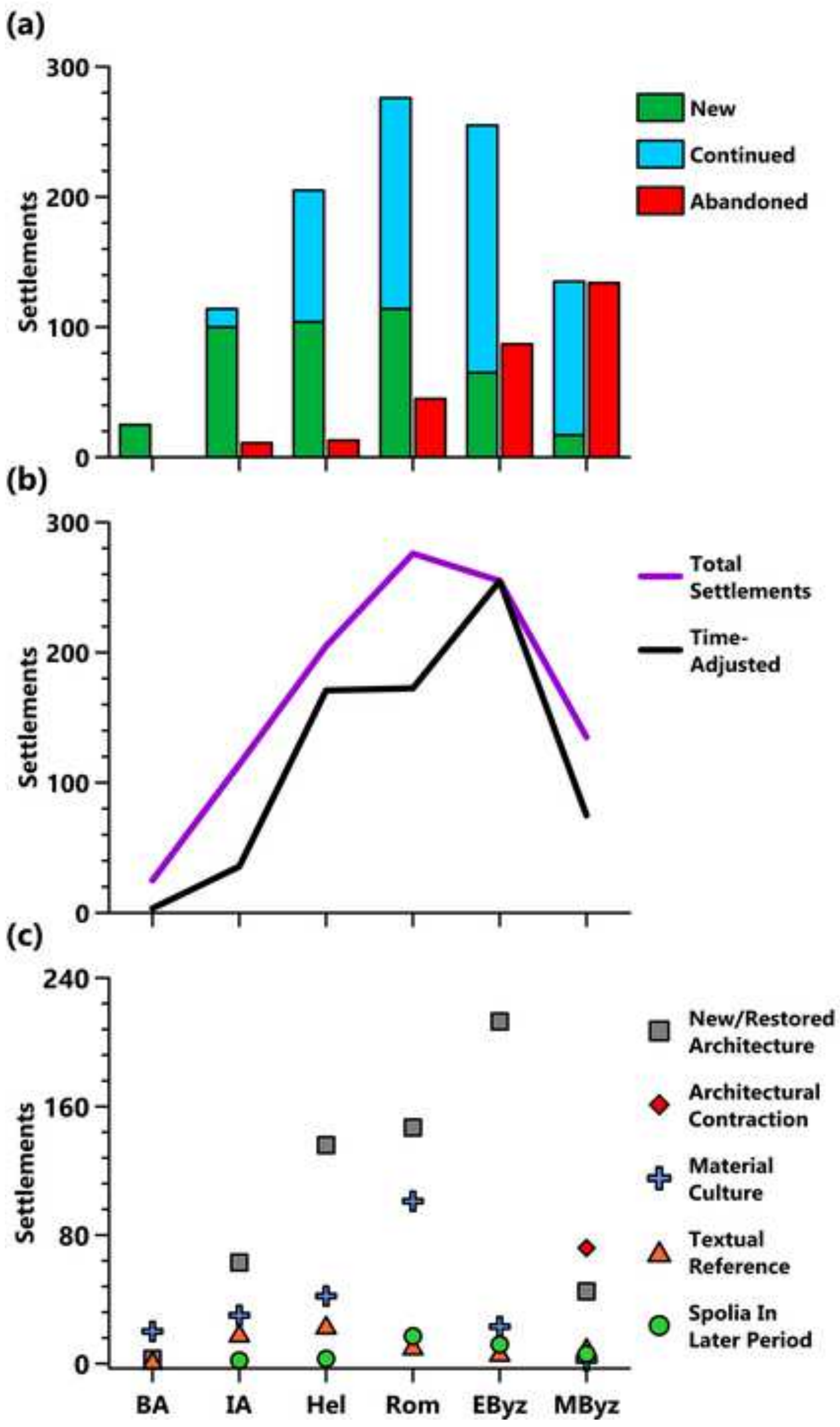

Figure 3

Click here to access/download;Figure;Fig 3 Maps_TIFF.tif

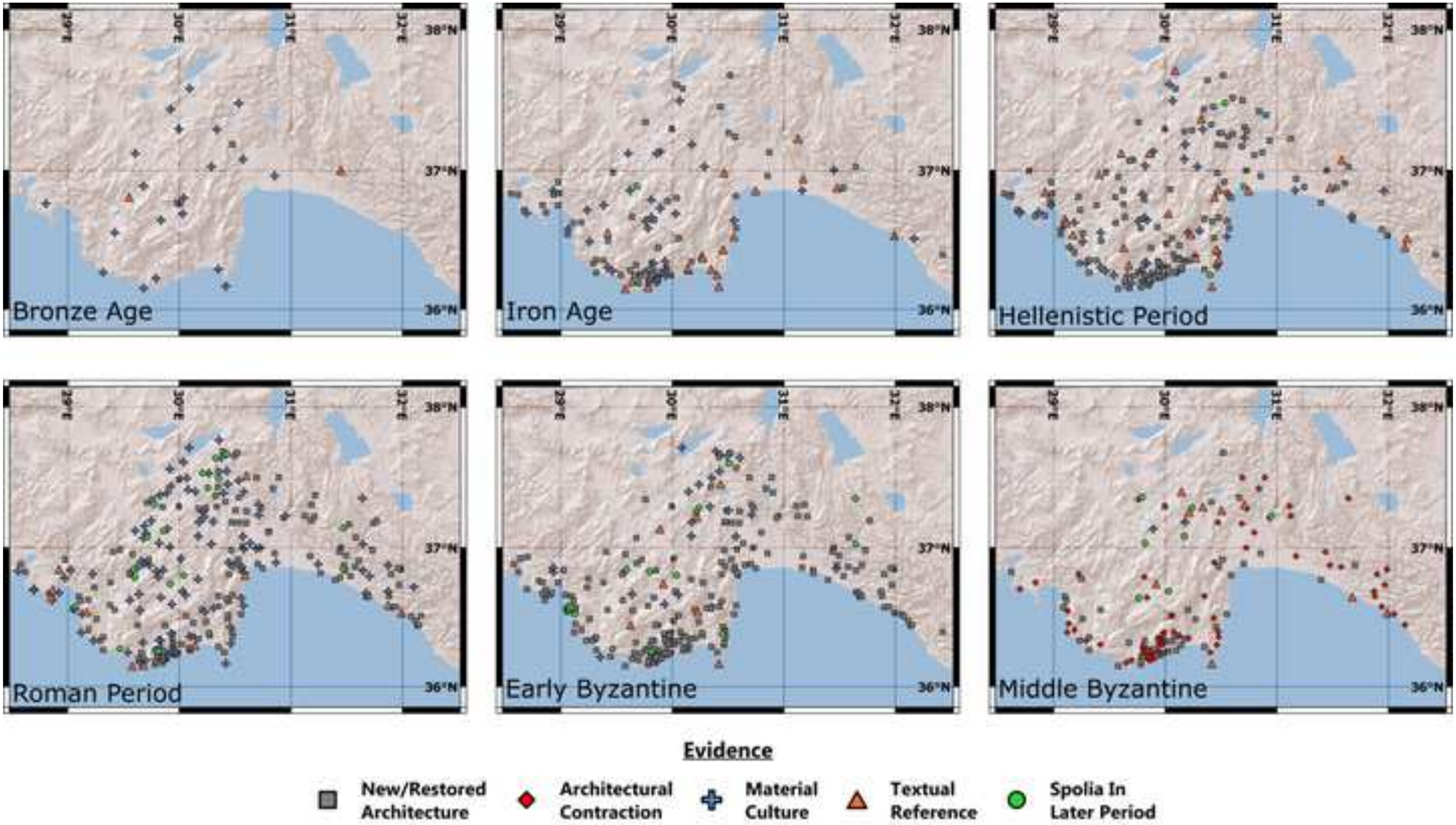

Figure 4

Click here to access/download;Figure;Fig 4 Mosaic v3_TIFF.tif

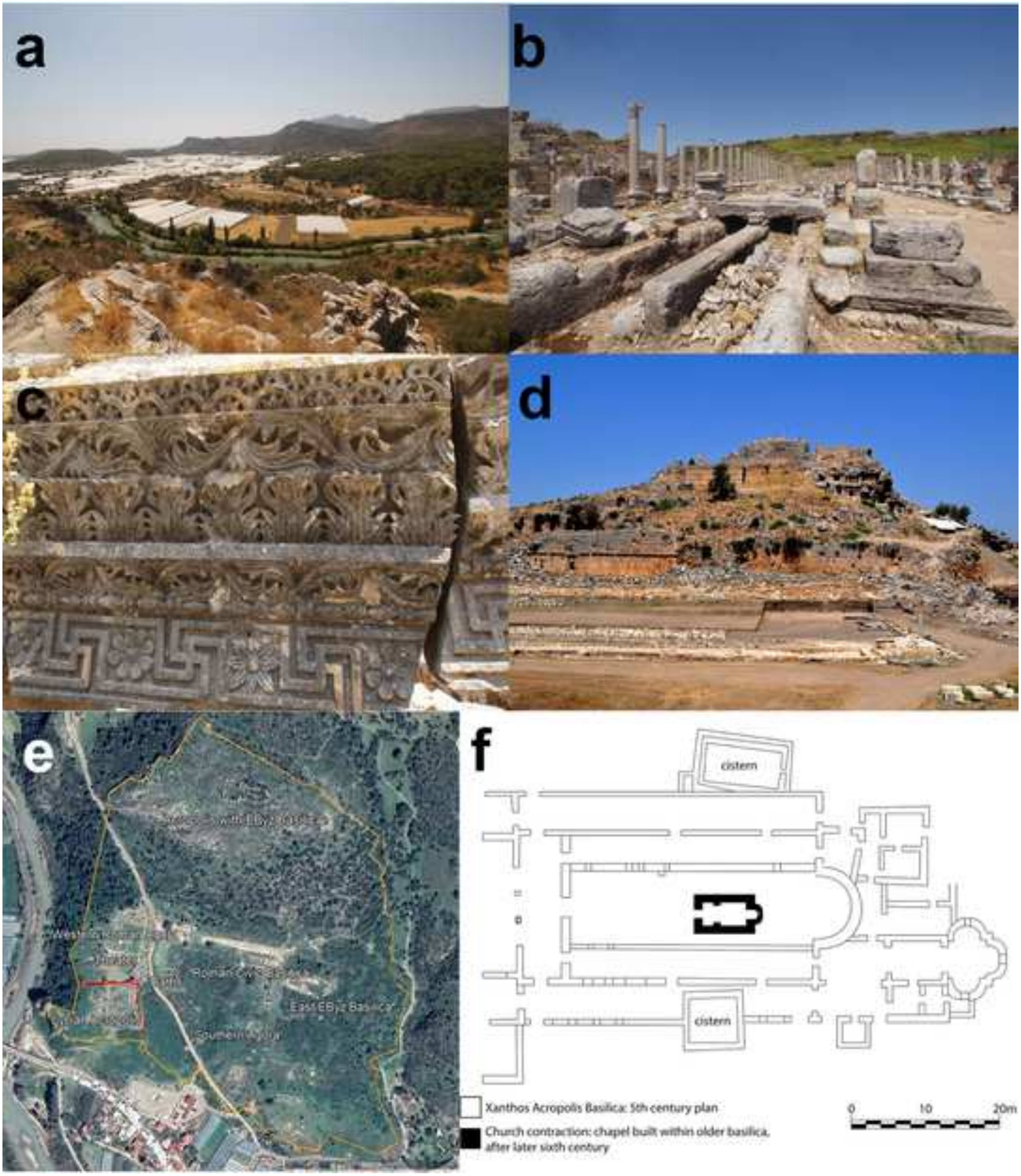

Figure 5

Click here to access/download;Figure;FIG5EL~1.TIF

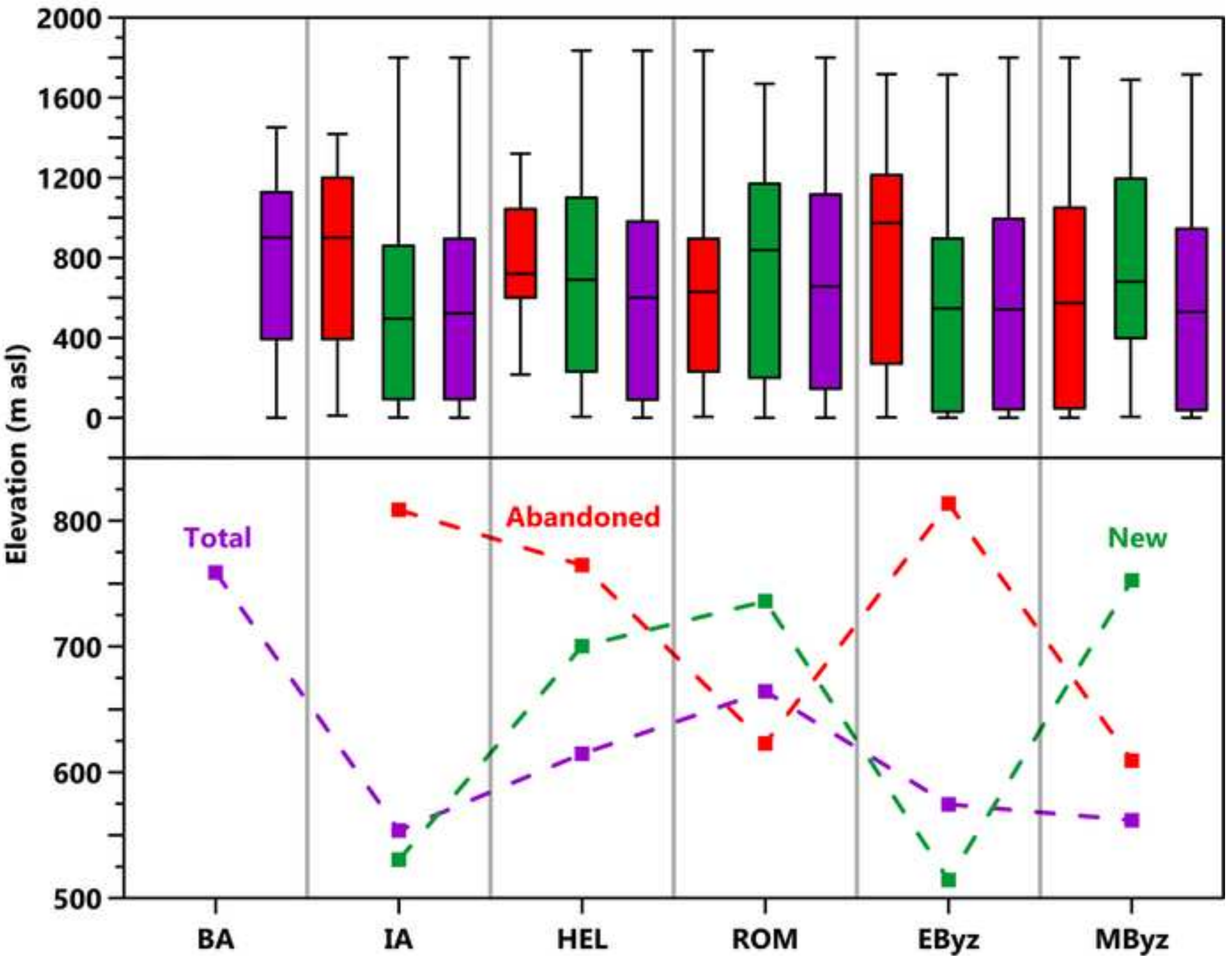

Figure 6

Click here to access/download;Figure;FIG6PA~1.TIF

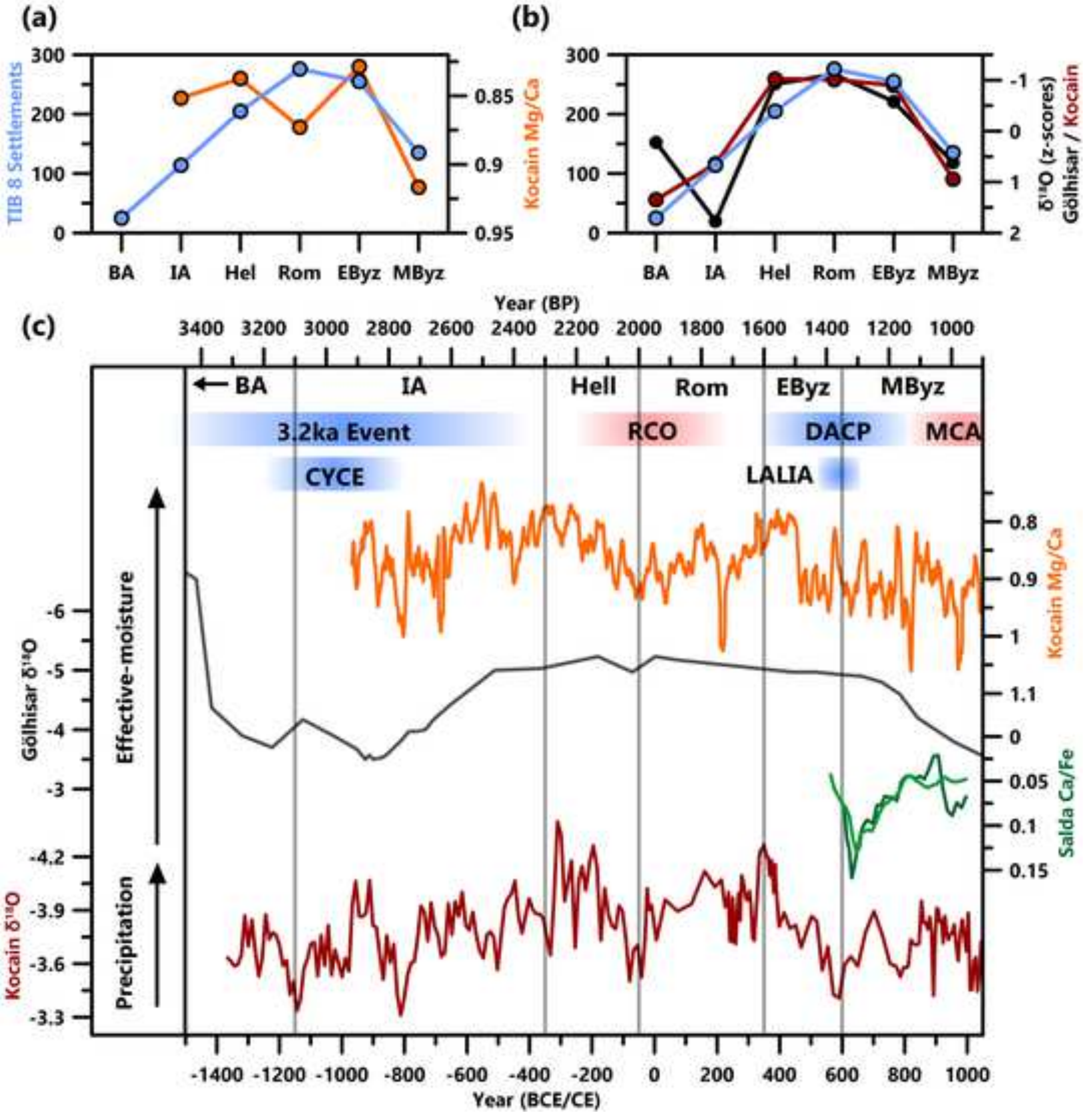

Figure 7

Click here to access/download;Figure;Fig 7 Surveys_TIFF.tif

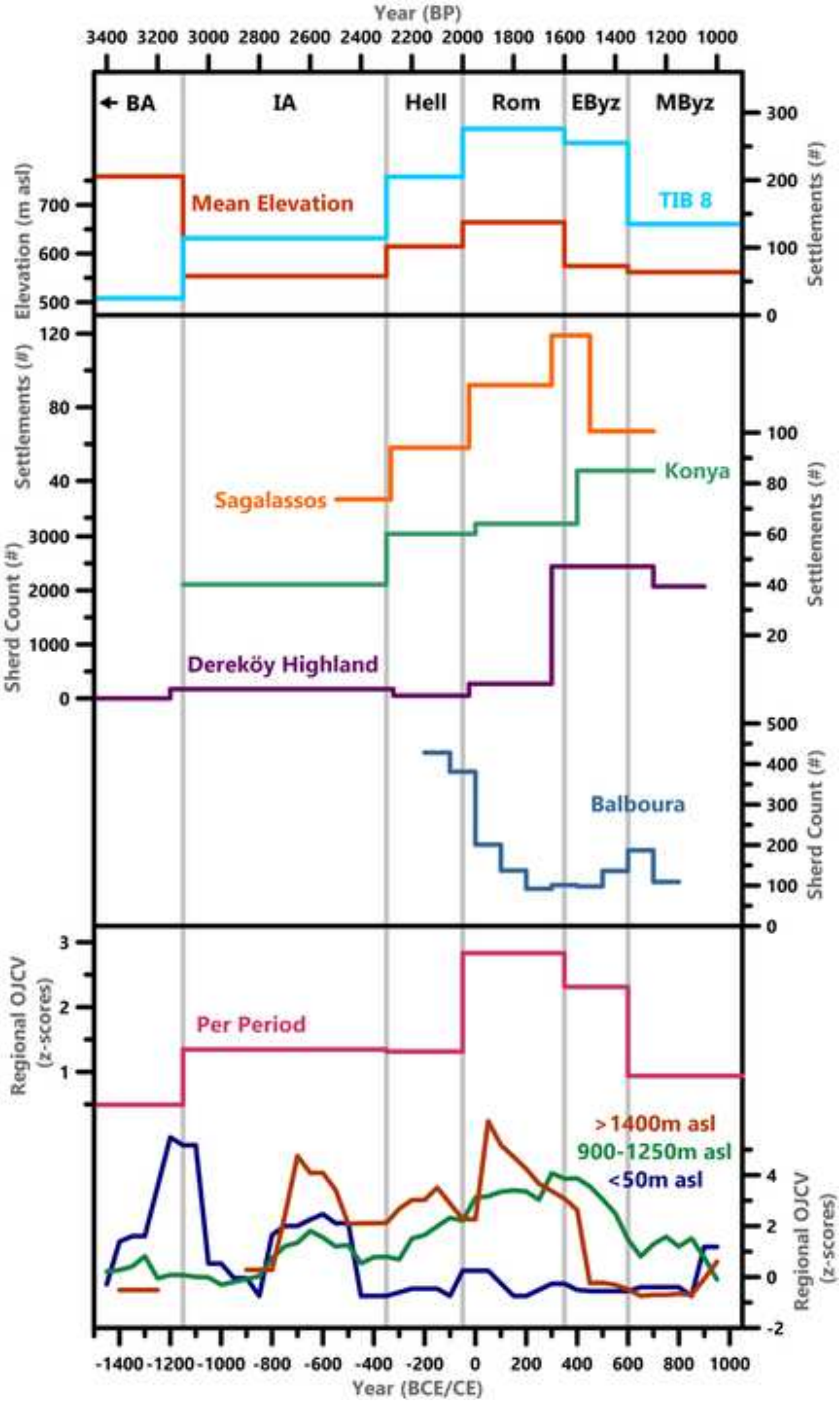

Supporting Table S1

Click here to access/download
Supporting Information
Table S1 - Settlement Dataset.xlsx

Supporting Table S2

Click here to access/download
Supporting Information
Table S2 - Palaeo-data.xlsx

Supporting Table S3

Click here to access/download
Supporting Information
Table S3 - Additional Byzantine Evidence.xlsx

Supporting Figure S1

Click here to access/download
Supporting Information
Fig S1 Resolutions_TIFF.tif

Supporting Figure S2

Click here to access/download
Supporting Information
FIGS2P~1.TIF

Supporting Figure S3

Click here to access/download
Supporting Information
FIGS3C~1.TIF

Supporting Figure S4

Click here to access/download
Supporting Information
Fig S4 OJCV Values_TIFF.tif
