# Supplementary material for: Settlement, environment, and climate change in SW Anatolia: Dynamics of regional variation and the end of Antiquity
Source: PLoS One. 2022 Jun 27;17(6):e0270295. doi: 10.1371/journal.pone.0270295 (PMC9236232; doi:10.1371/journal.pone.0270295)
Supplement: Attachment — Submitted filename: Response to Reviewers.docx [file pone.0270295.s010.docx]

Response to Reviewers - Settlement, environment, and climate change in SW Anatolia: dynamics of regional variation and the end of Antiquity.

“Revision required [PONE-D-22-00644] - [EMID:2e1a99188915b39d]”

**Journal Requirements –**

1. PLOS ONE Style requirements

Manuscript has been re-formatted according to PLOS One guidelines

2. Permits for work

No permits required, statement added to end of methodology: “No permits were required for the described study, which was exclusively desk-based and complied with all relevant regulations.”

3. Inclusivity questionnaire

Included in re-upload

4. Amended funding statement

Included in cover letter

5. Data availability statement

Included in cover letter

6. Possibly copyrighted map images

Maps have now been updated to be based on the ASTER Global DEM v3 which is cited in figure captions.

**Reviewer #1 –**

Changes to introduction – moved the end of the introduction to the conclusion

Indicate typology of satellite images and software GIS used – satellite images from Google Earth used for identification, but not included in the figures. QGIS software now mentioned in methods section and in relevant figure captions.

Non-climatic reasons referenced throughout – relevant non-climatic reasons are referenced throughout the text, the factors mentioned at the end of the discussion are only relevant for the final section.

Non-climatic factors figure – New figure for the E-Byz to M-Byz transition added (Fig 8)

Paleoclimate figure split into 3 parts – this figure is separated to emphases a few points in the first part of the discussion about comparing paleoclimate to archaeological data. (a) and (b) compare the period-averaged climate data (which we say can be misleading) to the settlement data (which is lower resolution). (a) and (b) are separated to show that the correlation between precipitation proxies and settlements can be misleading, considering that effective moisture (evidenced in Mg/Ca ratios) is a better predictor of agricultural productivity. No changes were made to the figure, the reason for the split is explained in the paragraph preceding “A Roman Climatic Optimum?”

Shortening the paper – we don’t think anything can be removed from the paper without weakening the arguments made. The “Data Critique” section could be removed, or added to the supplementary; however, reviewer #1 likes this section and we agree it should remain included in the body of the text. Furthermore, this kind of honest critique section is normally confined to the supplementary so that it isn’t seen – we want to be as transparent as possible with our dataset.

Mistakes to table and figure numbers – corrected.

Caption of figure 2 to describe “New” etc. – This is now included in the caption for Table 1 (settlement metadata), which comes long before Figure 2 in the text. This statement could also be added to the figure caption if needed:

“New” settlements are those with evidence in the period, but no evidence in the preceding period; “Continued” settlements are those with evidence in the period and preceding period; “Abandoned” settlements are those with no evidence in the period, but evidence in the preceding period.”

Figure 3 (now Fig 4) scale not readable – corrected by changing colour and shape of symbols, also made the figure fit better on an a4 page so that the individual maps are larger.

Figure 4e (now figure 7e due to re-ordering) line thickness – red and orange lines now as thick as they can be without obscuring the image. Writing is also larger and scale-bar more obvious with white background.

**In Paper Comments –**

Throughout – corrected all text to be consistently US-English
Re-ordered figures to align correctly with their first mention in-text and corrected all links to figures.

Page 1 – added new affiliation and email for MJ Jacobson

Page 2 – added broad references for low availability of proximate records and corrected brackets

Page 4 – Moved the end of the introduction to the conclusion, as recommended by reviewer.

Methods – Page 5-10 – whole section re-ordered for clarity, with table order and links adjusted accordingly.

Page 7 – Table 1 – explanation of “New”, “Continued” and “Abandoned” settlements included here as this is the first instance of their mention.

Page 8 – Table 2 – ages of periods added

Page 9-10 – table 3 – explained measures of resolution better.

Page 11 – changed sentence to read “For many settlements, particularly **in** rural **locations**,…”

Page 11 – changed Turkish spelling of “Tarsus” to “Tarsos”

Page 12 – changed fractions to %s

Page 23-24 – corrected Turkish spelling of Oludeniz to Ölüdeniz.

Page 24 – corrected Turkish spelling of Sogut to Söğüt

Page 25 & 26 – corrected Turkish spelling of Gemiler Adasi to Gemiler Adası

Page 28 – corrected In after “…Byzantine periods:” to in

Page 33 – changed Turkish spelling of “Tarsus” to “Tarsos”

Page 33 – added part of the conclusion which was moved from introduction (Page 4)

Biblio – corrected some references
